# Diversity and molecular network patterns of symptom phenotypes

**DOI:** 10.1038/s41540-021-00206-5

**Published:** 2021-11-30

**Authors:** Zixin Shu, Jingjing Wang, Hailong Sun, Ning Xu, Chenxia Lu, Runshun Zhang, Xiaodong Li, Baoyan Liu, Xuezhong Zhou

**Affiliations:** 1 Institute of Medical Intelligence, School of Computer and Information Technology, Beijing Jiaotong University, Beijing, 100063 China; 2 The First Affiliated Hospital of Henan University of Chinese Medicine (Co-construction Collaborative Innovation Center for Chinese Medicine and Respiratory Diseases by Henan, Henan University of Chinese Medicine), Zhengzhou, 450046 China; 3 Hubei Provincial Hospital of Traditional Chinese Medicine (Affiliated Hospital of Hubei University of Traditional Chinese Medicine, Hubei Academy of Traditional Chinese Medicine), Wuhan, 430061 China; 4 Guang’anmen Hospital, China Academy of Chinese Medical Sciences, Beijing, 100053 China; 5 China Academy of Chinese Medical Sciences, Beijing, 100700 China

**Keywords:** Molecular biology, Signs and symptoms

## Abstract

Symptom phenotypes have continuously been an important clinical entity for clinical diagnosis and management. However, non-specificity of symptom phenotypes for clinical diagnosis is one of the major challenges that need be addressed to advance symptom science and precision health. Network medicine has delivered a successful approach for understanding the underlying mechanisms of complex disease phenotypes, which will also be a useful tool for symptom science. Here, we extracted symptom co-occurrences from clinical textbooks to construct phenotype network of symptoms with clinical co-occurrence and incorporated high-quality symptom-gene associations and protein–protein interactions to explore the molecular network patterns of symptom phenotypes. Furthermore, we adopted established network diversity measure in network medicine to quantify both the phenotypic diversity (i.e., non-specificity) and molecular diversity of symptom phenotypes. The results showed that the clinical diversity of symptom phenotypes could partially be explained by their underlying molecular network diversity (*PCC* = *0.49, P*-value = *2.14E-08*). For example, non-specific symptoms, such as chill, vomiting, and amnesia, have both high phenotypic and molecular network diversities. Moreover, we further validated and confirmed the approach of symptom clusters to reduce the non-specificity of symptom phenotypes. Network diversity proposes a useful approach to evaluate the non-specificity of symptom phenotypes and would help elucidate the underlying molecular network mechanisms of symptom phenotypes and thus promotes the advance of symptom science for precision health.

## Introduction

Symptom phenotypes (i.e., symptoms and signs), one of the main clinical manifestations of disease conditions, that could be obtained by human natural perception and cognition abilities, play a vital role for medical visiting, clinical diagnosis, and disease treatment. It has been well-recognized that exploring the clinical patterns and their underlying molecular mechanisms of symptom phenotypes would contribute significantly to nursing science and precision medicine^1,2^. However, non-specificity (or diversity) is one of the main obstacles to fully utilize the symptom phenotypes for both diagnosis and treatment. In particular, it has been estimated that Medically Unexplained Symptoms such as tiredness, dizziness, and headache^3^, which are actually the first part of manifestations in early stage of disease, account for up to 49% of all general practice consultations and high healthcare cost^4^. This means there has no specified pathology to sufficiently reveal and explain the persistent bodily complaints^5^.

Furthermore, due to the network pathological mechanisms of clinical manifestations, symptoms tend to occur together clinically to form symptom clusters^6^ across different chronic disease condition^7^, which would be more specific and meaningful for diagnosis and treatment. Therefore, the assessment of symptom clusters has been recognized as a promising research task for symptom science. For example, the identification of the typical symptom clusters and their underlying mechanisms, such as depression and pain^8^, have promoted the understanding of mental disorders and better treatment. In addition, network medicine approach^9^ to investigate the interconnection of symptoms in mental disorders has emerged as one of the most popular investigation methods in the field of psychometrics^10^.

**Fig. 1:**
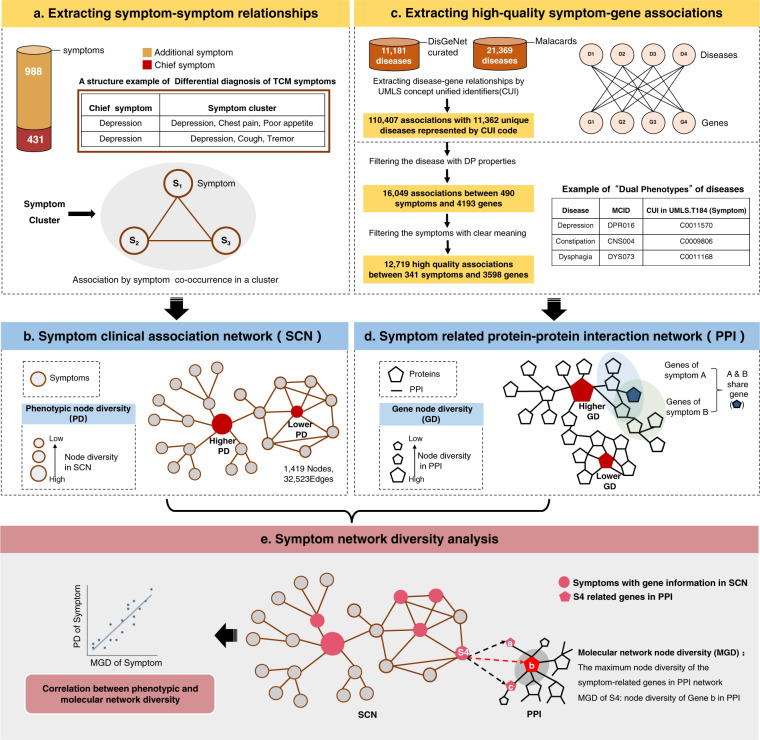
Quantifying the phenotypic and molecular network diversity of symptom phenotypes. **a** Curation of symptom-symptom relationships. The associations between symptoms are based on their co-occurrence in a symptom cluster of a textbook named *differential diagnosis of traditional Chinese medicine symptom*. **b** Constructing symptom clinical association network. The nodes represent symptoms and size reflects the phenotypic diversity in network. **c** Extracting high-quality symptom-gene associations. **d** Integrating both symptom-gene associations and protein–protein interaction (PPI) database to obtain molecular network diversity of symptom phenotypes. **e** The main steps of symptom network diversity analysis. We measured symptom diversity from both phenotypic and molecular network contexts.

However, although it is vital there is no work to quantify the diversity of symptom phenotypes in the context of clinical settings and their underlying molecular networks, largely because of the lack of high-quality symptom-gene associations and clinical symptom co-occurrence data. Here, we extracted symptom co-occurrences from clinical textbooks to construct phenotype network of symptoms with clinical co-occurrence and incorporated high-quality symptom-gene associations^11^ and protein–protein interactions to explore the molecular mechanisms of symptom phenotypes^12^. Furthermore, we adopted a well-established measure in network medicine^13^ to quantify both phenotypic and molecular diversity of symptom phenotypes (Fig. 1).

## Results

### High-quality symptom-gene associations

To obtain the high-quality symptom gene associations, we utilized the phenomenon of some “Dual Phenotypes” (DP)^14^, such as obesity, fever, and insomnia, which are not only regarded as diseases, but also as symptoms in clinical settings. The associated genes of symptoms can be directly derived from the disease–gene associations by filtering the disease with DP properties. In order to identify these kinds of phenotype terms, we filtered an integrated phenotype–genotype associations (PGA) dataset by limiting the semantic types of Unified Medical language System (UMLS) concepts as T184^15^, which resulted in 16,049 associations between 490 symptoms with concept unified identifiers (CUI) code and 4193 genes (see Methods). In fact, these concepts including syndromes (e.g., kearn sayer syndrome), signs (e.g., abnormal reflexes), laboratory tests (e.g., leukopenia) and diseases (e.g., edema lung). Therefore, we manually reviewed and removed symptoms without clear meaning under the guidance of medical to ensure the accuracy of results (Supplementary Table 2). Finally, we obtained 12,719 high-quality symptom–gene associations between 341 symptoms and 3598 genes.

**Fig. 2:**
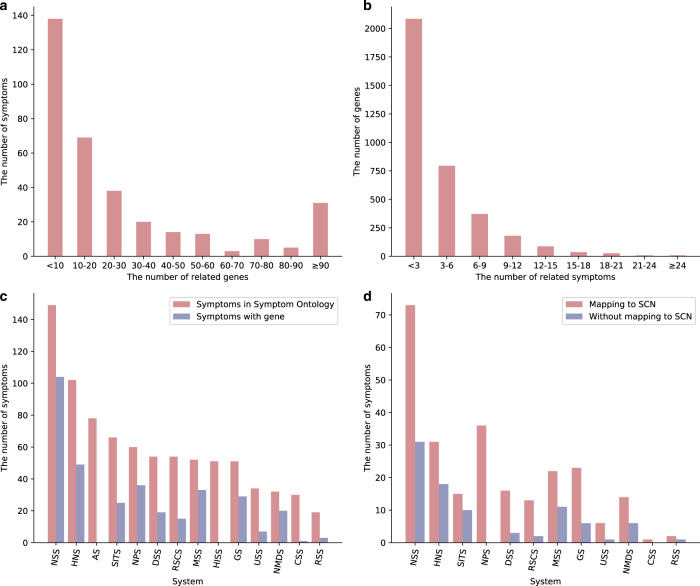
The basic statistics of high-quality symptom-gene associations. **a** The distribution of symptom-related genes. **b** The distribution of gene-related symptoms. **c** The distribution of related system categories of symptoms. We compared the class information of symptoms with gene information to the ontology. **d** Mapping distribution of symptoms with genetic information to SCN. We compared the different system categories of symptoms with genes information grouped by mapping to SCN. The full name of the system: NSS Nervous System Symptom, HNS Head and Neck Symptom, AS Abdominal Symptom, SITS Skin and Integumentary Tissue Symptom, NPS Neurological and Physiological Symptom, DSS Digestive System Symptom, RSCS Respiratory System and Chest Symptom, MSS Musculoskeletal System Symptom, HISS Hemic and Immune System Symptom, GS General Symptom, USS Urinary System Symptom, NMDS Nutrition, Metabolism, and Development Symptom, CSS Cardiovascular System Symptom, RSS Reproductive System Symptom.

Here, we found there are 37.30 related genes on an average per symptom and 3.53 related symptoms for a single gene. More specifically, 60% symptoms have less than 20 associated genes (Fig. 2a); however, there still exist several symptoms with hundreds of genes, such as obesity (560 genes) and convulsion (673 genes), which indicate the underlying complex pathophysiology and comorbidities of these symptom phenotypes^16–18^. On the other side, over 50% genes have less than 3 associated symptoms, whereas some genes, such as PRNP, PSEN1, MAPT, GBA, and MECP2 are associated to >20 symptoms (Fig. 2b).

Furthermore, we mapped 341 symptoms to 14 systems or categories according to Symptom Ontology (SYMP) with the principles of the OBO Foundry^19^. The SYMP standard ontology (https://www.ebi.ac.uk/ols/ontologies/symp/terms) was developed in 2005 at the Institute for Genome Sciences (IGS) at the University of Maryland and contain more than 900 symptoms in 2020. Despite the limited number of our symptom terms, it covers almost all system categories, which of the large number of symptoms belong to the nervous system (Fig. 2c).

### Clinical diversity of symptom phenotypes

**Fig. 3:**
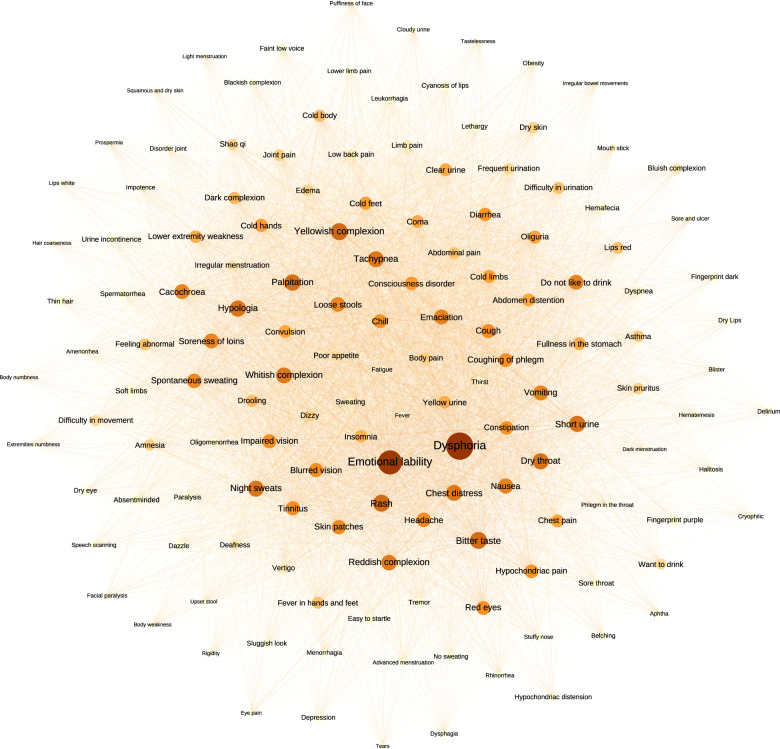
Construction of symptom clinical association network(SCN). The nodes indicate the symptoms and interconnecting edges in SCN represent the clinical co-occurrence. Node size and color reflected the diversity of symptom phenotypes in SCN (a high diversity is represented by large size node and deep orange color node). Here, filtering the node and related edges of symptom phenotypic diversity value <60 in the network and remaining 144 nodes and 6894 edges are visualized.

To measure the symptom diversity in the context of network, we first constructed a symptom clinical association network (SCN) using 2381 records of symptom clusters curated from a well-recognized textbook named *differential diagnosis of traditional Chinese medicine symptoms* (DDTS)^20^, which resulted in a network with 1419 nodes (symptoms) and 32,523 links. In SCN, the symptoms with higher phenotypic diversity (PD) and phenotypic degree (PE), such as neurological and physiological symptoms (e.g., dysphoria, PD: 100.32, PE: 623), respiratory system symptoms (e.g., chest distress, PD: 89.24, PE: 381), and digestive system symptoms (e.g., diarrhea, PD:84.24, PE: 230) which may involve in a various of diseases (Fig. 3). For example, for diarrhea^21^ accompanied with abdominal pain, fever, or gastrointestinal bleeding, it would suggest inflammatory diseases. For another diarrhea phenotype with symptoms of fatigue, cough, and fever, it might relate to virus infectious diseases, such as the severe acute respiratory syndrome coronavirus 2^22^. Other top ranked symptoms, such as night sweats (PD:89.44, PE:282) and difficulty in urination (PD:80.93, PE:177) (Table 1) would tend to occur as complications in a critical condition. However, the symptoms with low diversity, such as nail symptoms (e.g., flat nails, PD:0.95, PE: 2) and feet symptoms (e.g., digit fester, PD:3.57, PE:8), tend to be local clinical manifestations.

**Table 1:** Quantifying the diversity of symptom phenotypes in SCN (including the top 50 symptoms sorted by the phenotypic diversity in SCN).

Symptom	PD^a^	PE^b^	Symptom	PD	PE
Dysphoria	100.33	623	Cough	85.76	321
Emotional lability	99.34	632	Blurred vision	85.73	344
Yellowish complexion	91.26	327	Impaired vision	85.65	345
Rash	91.15	367	Coughing of phlegm	85.37	264
Bitter taste	90.98	330	Red eyes	85.26	251
Palpitation	90.07	366	Chill	85.06	502
Short urine	89.81	319	Hypochondriac pain	84.71	225
Dry throat	89.75	388	Constipation	84.65	539
Hypologia	89.68	253	Diarrhea	84.24	230
Night sweats	89.44	282	Consciousness disorder	84.09	351
Chest distress	89.24	381	Cold hands	83.83	254
Tachypnea	89.22	381	Cold feet	83.63	250
Whitish complexion	89.17	412	Cold limbs	83.43	349
Reddish complexion	88.65	451	Oliguria	83.25	220
Nausea	87.75	301	Chest pain	83.12	197
Vomiting	87.54	311	Convulsion	83.01	284
Do not like to drink	87.38	243	Abdomen distention	82.97	457
Emaciation	87.36	363	Coma	82.85	261
Cacochroea	87.31	252	Clear urine	82.84	213
Soreness of loins	87.25	360	Fullness in the stomach	82.80	244
Loose stools	86.94	432	Yellow urine	82.53	507
Spontaneous sweating	86.69	229	Insomnia	82.37	514
Headache	86.29	394	Lower extremity weakness	82.33	230
Skin patches	86.27	249	Dark complexion	81.62	200
Tinnitus	85.89	343	Cold body	81.51	195

^a^PD means the symptom phenotypic diversity in SCN; ^b^PE means the symptom phenotype degree in SCN.

### Molecular network diversity of symptom phenotypes

**Fig. 4:**
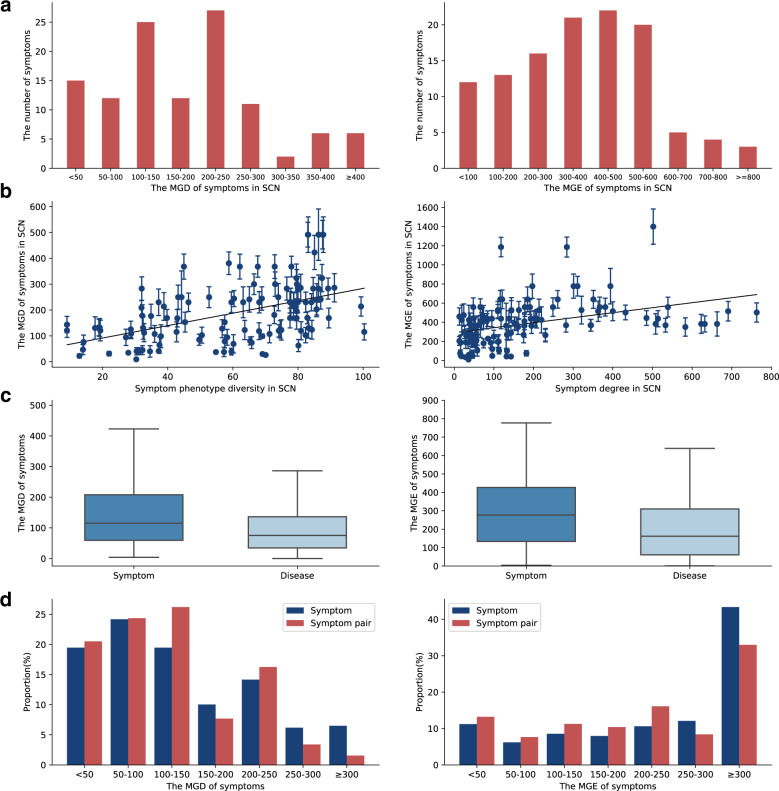
Symptom network diversity analysis. **a** The MGD and MGE distribution of symptoms in SCN. **b** Correlations of the symptom diversity between phenotypic and molecular networks. **c** Compared the MGD and MGE distribution of symptoms and diseases. On each box, the central mark indicates the median, the bottom and top edges of the box indicate the 25th and 75th percentiles, respectively. The whiskers extend to the most extreme data represent the minimum and maximum value. **d** Compared the MGD and MGE distribution of symptoms and symptom pairs.

To explore the underlying molecular mechanisms of symptom phenotypic diversity, we mapped 252 (73.90%) English terms with associated genes into 116 Chinese terms in SCN (see Methods, Supplementary Table 1), including neurological and physiological symptoms (e.g., night sweats) and general symptom (e.g., chill). 89 (26.10%) symptoms not mapped are mostly from nervous system symptoms (e.g., echo speech), head and neck symptoms (e.g., conjunctiva inflammation), and musculoskeletal system symptoms (e.g. gait ataxic) (Fig. 2d). Next, we attempt to calculate the maximum node diversity and degree of the symptom-related genes in protein–protein interactions (PPI) network^23^ to represent molecular network diversity (MD) of symptom phenotypes (see Methods). The maximum gene diversity (MGD) of 116 symptoms range from 9.12 to 491.39, and ~45% of symptoms had MGDs greater than 200. The maximum gene degree (MGE) of symptoms range from 10 to 1400, and only 10% symptoms had a value greater than 600 (Fig. 4a, b) (Table 2).

**Table 2:** Quantifying the molecular network diversity of symptom phenotype in SCN (including the top 50 symptoms sorted by the molecular network diversity in SCN).

Symptom	MGD^a^	MGE^b^	Symptom	MGD	MGE
Convulsion	491.39	1186	Brash	249.61	410
Vomiting	491.39	777	Thin hair	249.61	410
Nausea	491.39	777	Voice hoarseness	249.61	410
Chest pain	491.39	777	Nail thinness	249.61	410
Headache	491.39	777	Rigidity	244.27	515
Chill	422.73	1400	Fatigue	244.27	515
Dyscalculia	380.77	1186	Fever	241.09	502
Obesity	367.89	639	Loose stools	241.09	502
Deafness	367.89	558	Cough	241.09	527
Body weakness	367.89	527	Skin pruritus	233.94	362
Decreased hearing	367.89	558	Difficulty in movement	229.93	437
Tremor	367.89	527	Consciousness disorder	229.93	639
Emaciation	323.43	515	Failure to thrive	229.93	437
Edema	323.43	527	Disorder joint	229.93	437
Speech scanning	299.95	639	Jaundice	229.93	437
Amnesia	299.95	639	Limb pain	229.93	437
Depression	299.95	522	Joint pain	229.93	437
Rash	286.07	558	Insomnia	229.93	437
Body pain	285.63	444	Low back pain	229.93	437
Abdominal pain	285.63	515	Joint swollen	229.93	437
Constipation	282.94	558	Aphtha	229.93	437
Dyspnea	282.94	558	Poor appetite	213.66	350
Skin patches	282.94	558	Anorexia	213.66	350
Delay language	282.94	558	Emotional lability	213.66	382
Tachypnea	282.94	558	Blindness	208.68	350

^a^MGD means the maximum node diversity of the symptom-related genes in PPI network; ^b^MGE means the maximum node degree of the symptom-related genes in PPI network.

Here, we calculated the Pearson correlation coefficient (PCC) to find the relationships of phenotypic and molecular diversity of these symptoms. The result showed that there exists a positive correlation between the two measures (PD and MGD: *PCC* = *0.49*, *P*-value = *2.14E-0*8; PE and MGE: *PCC* = *0.39*, *P*-value = *1.55E-05*) (Fig. 4b). This means that symptoms occurred in more symptom clusters might tend to held higher diverse underlying molecular networks. For example, we found depression have rather high MGD (299.95), which actually is derived from the high diversity of the related gene: MAPK1 in PPI network. MAPK1 as one of the important regulated gene in the mTOR signaling pathway which plays an important role in synaptic plasticity in Alzheimer’s disease and relate to the depression disorder as well as functioning of the immune system^24,25^. It is similar for obesity, which has high MGD (367.89) and is considered both as complicated chronic disease condition and symptom with a major negative impact on human health. Since one of the vital obesity genes: AKT1 has the high node diversity (367.89) in PPI network, which at molecular level not only mediated type II muscle growth and thus led to the reversible reduction of fat mass, but also have a direct role on cancer and hearing loss^26–29^.

**Fig. 5:**
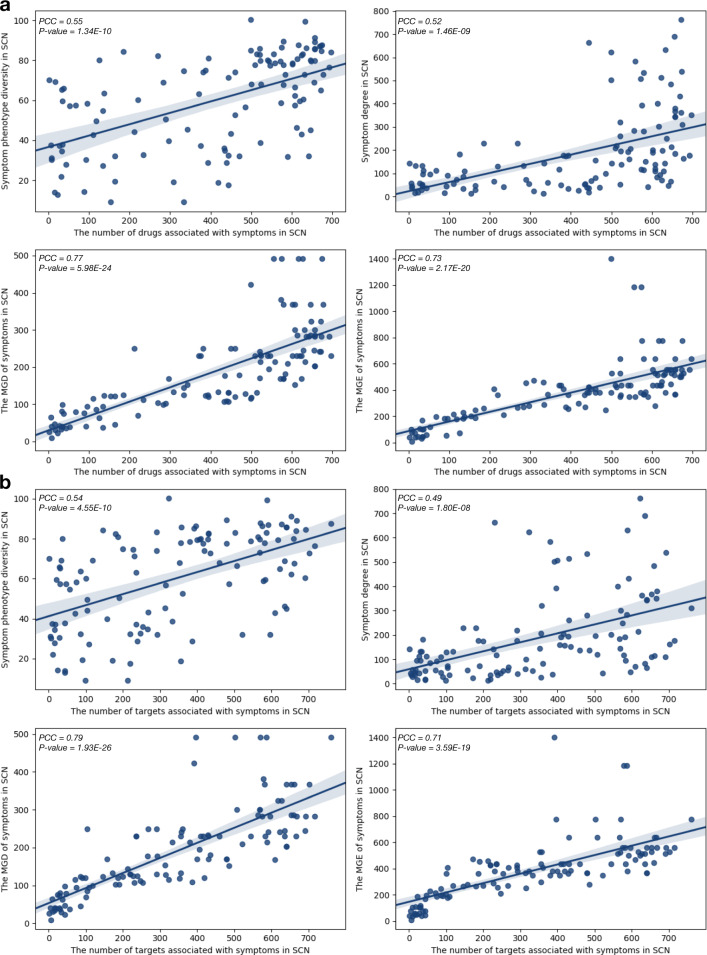
Correlations of the symptom network diversity and related drug-targets diversity. **a** Correlations between the symptom diversity (phenotypic and molecular networks) and the number of related drugs. **b** Correlations between the symptom diversity (phenotypic and molecular networks) and the number of related drug-targets.

To further validate and detect the potential applications of symptom diversity for drug development, we curated 948 drugs and their 1451 drug targets from the DrugBank database^30^ and calculated the correlations between symptom diversity to the number of drug targets located in the neighborhoods of symptom genes in the PPI network. We would expect that drugs tend to regulate symptom by directly targeting symptom genes or the neighbors of symptom genes, the similar principle of which has been used for various related studies^31^. After obtaining the related drug targets associated with 116 symptoms in the 1^st^ order PPI interactions, we found that there actually exists a strong positive correlation between the number of drug targets and the MGD of symptoms (*PCC* = *0.79*, *P*-value = *1.93E-26*, Fig. 5b). This is similar for phenotypic network diversity (*PCC* = *0.54*, *P*-value = *4.55E-10*, Fig. 5b). The results indicate that symptoms with higher diversity in the clinical settings may tend to have higher number of drug targets to regulate the underlying molecular mechanisms of symptoms. Symptoms with higher drug target number (DTN) also have higher phenotypic diversity, such as dysphoria (DTN: 323), insomnia (DTN: 431), and vomiting (DTN: 761). For example, about 10 categories of drugs are associated with insomnia, including antihistamine (e.g., doxylamine^32^), anxiolytics (e.g., etizolam^33^), and antipsychotics (e.g. melperone^34^), which affect GABA-A, D2 dopaminergic and 5HT2A serotonergic and other receptors to treat insomnia. Thus, the symptoms with more clinical diversities would have the potential to be induced and treated by more drugs that target the related genes in their PPI neighborhoods. Furthermore, it is also interesting and important to validate whether the trend is also held for diseases. Therefore, using the integrated disease-gene associations with 179,307 records (12,563 diseases and 18,189 genes), we further investigate the correlation between disease diversities (i.e., in terms of its underlying molecular network) and the number of their drug targets by additional calculations. We found that there exactly exists a strong positive correlation between the number of drug targets and the MGD of diseases *(PCC* = *0.77, P*-value < *4.9E-324)*. This is similar for the number of drugs *(PCC* = *0.74, P*-values < *4.9E-324)*. These results indicate that diseases with higher diversity in the molecular network may tend to have higher number of drug targets (Supplementary Fig. 1).

### Molecular network diversity (symptom vs disease phenotypes)

Traditional clinical diagnosis often relied on symptom manifestations, which would be more directly be observed in patients’ daily life and thus convenient for clinical management. However, similar symptom phenotypes always involved in different disease conditions, which would propose substantial obstacles for clinical diagnosis and treatment. Due to the more specific mechanisms of disease phenotypes, changing from symptom-based diagnosis to disease-based diagnosis is the main contribution of modern disease taxonomy and biomedical science^35–39^. To validate the advantages of disease diagnosis, we utilized the disease–gene associations from MalaCards to similarly calculate the MD for 12,563 disease phenotypes. We found that disease phenotypes tend to have lower diversity than those of symptom phenotypes in terms of MGD (median: 75.39 vs 115.16, *P*-value = *9.03E-06*) and MGE (median: 162 vs 277, *P*-value = *4.58E-13*) (Fig. 4c, d). For example, the diseases, such as bronchitis (213.7), asthma (213.7), and rhinitis (153.3), have lower MGDs than those of cough (241.1), which are three typical causes of chronic cough^40,41^. The lower MD of disease phenotypes could partially explain their advantages as diagnostic schema in modern biomedicine.

### Clinical symptom clusters hold approach for specific molecular network mechanisms

To resolve the non-specificity of symptom phenotypes, many contemporary diagnoses owe their existence to symptom cluster which has been defined as two or more interrelated symptoms that present together and involve the similar etiology and pathophysiology, such as nephrotic syndrome, irritable bowel syndrome, and chronic fatigue syndrome^42–44^. Particularly, those symptom clusters with specific underlying common mechanisms have been accepted in clinical practice and frequently used by clinicians today^45–48^. Therefore, we would expect that the common molecular mechanisms involved in symptom clusters would propose an effective approach to reduce the high molecular diversity of a symptom phenotypes. To further validate this assumption, we obtained 1740 symptom pairs (as representations of symptom clusters) with the overlapping genes from SCN, which we found only 704 symptom pairs with symptom-gene association randomization (1740 vs 704, *P*-value = *3.07E-101*). This means that symptom pairs in SCN tend to have shared genes. Next, we obtained the MGDs of symptom pairs in terms of maximum node diversity of their shared genes. We found that symptom pairs tend to have significant lower MGD (median: 108.30 vs 115.16, *P*-value = *1.8E-04*) and MGE (median: 222 vs 277, *P*-value = *3.14E-08*) than those of single symptoms. Particularly, the proportions of MGD (4.94% vs 12.68%) and MGE (41.38% vs 55.46%) in high value (i.e., >=250) are lower in symptom pairs than in single symptoms (Fig. 4d). These results confirmed the significance of symptom clusters as a feasible solution to acquire specific understanding of disease conditions.

### Case study: insomnia symptom clusters

**Fig. 6:**
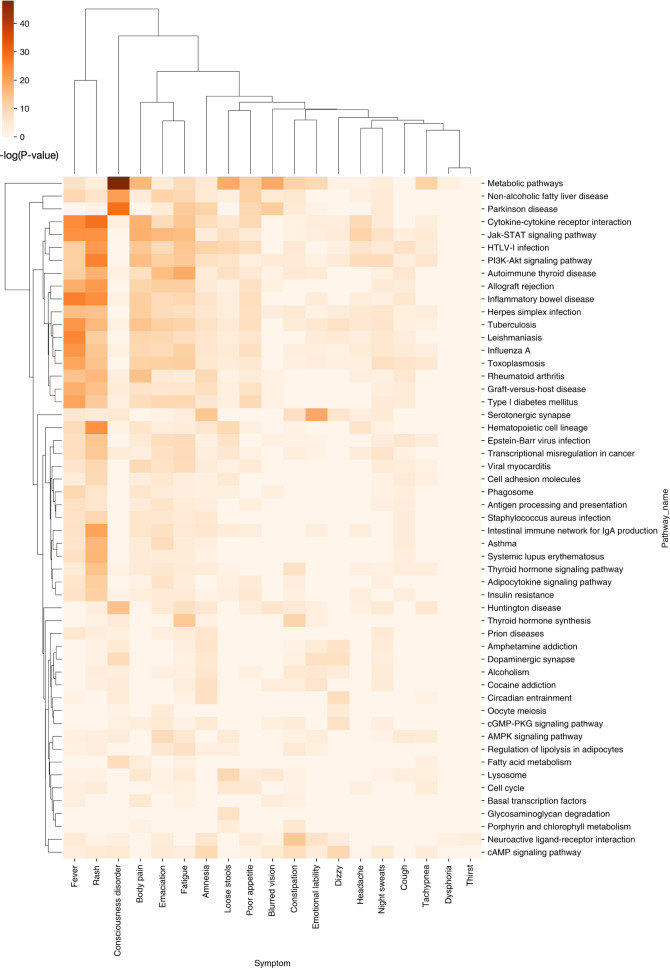
The overlapped pathways of insomnia symptom clusters. The enriched KEGG pathways is evaluated by *P*-value with <0.05.

Insomnia is a typical chronic disorder and symptom phenotypes that has both diverse underlying molecular mechanisms and can cause various psychiatric and physical health problems^49,50^. It has also been considered a strong risk factor of psychiatric illness, such as anxiety disorder, major depressive disorder^51^, and associated with many types of metabolic disease^52,53^, obstructive airway disease^54^, and cancer^55^. To investigate the underlying molecular mechanisms of specific symptom cluster, we identified 72 insomnia symptom pairs from 1740 clusters with overlapping genes. A total of 11 systems are involved in insomnia-related symptoms, which 36.2% of symptoms related to neurological and physiological systems, such as abdominal pain, amnesia, and dysphoria (Supplementary Fig. 2). We found 19 insomnia pairs with co-occurrence > =15 in DDTS, including the pairs of (insomnia, dysphoria), (insomnia, dizzy), and (insomnia, poor appetite) (Table 3). Moreover, we obtained the overlapped enriched KEGG^56^ pathways (*P*-value < 0.05) between these symptoms and insomnia to explore the shared molecular mechanisms of these insomnia pairs (see Methods). The number of enriched overlapped pathways of insomnia-related symptom pairs range from 1 to 49. Fever, fatigue, and amnesia have great overlapping pathways and co-occurrence with insomnia, which reflected the high diversity of these insomnia symptom pairs from both phenotype and molecular mechanisms (Table 3). For example, there are many reasons for insomnia patients with fever, such as influenza^57^, tuberculosis^58^, pneumonia^59^, tumors^60^, and neurological disorders^61^, which would be involved in various molecular pathways, including the immune system pathway (e.g., intestinal immune network for IgA production and intestinal immune network for IgA production), signal transduction pathway (e.g., cAMP signaling pathway and AMPK signaling pathway), and infectious disease pathway (e.g., Influenza A and Tuberculosis) (Fig. 6).

**Table 3:** The basic molecular features of insomnia symptom cluster (sorted by the co-occurrences).

Symptoms	Co-occurrences *n* (%)^a^	Overlap pathways *n* (%)^b^	Overlap genes
Emotional lability	160 (32.72)	26 (25.74)	NDST1, SLC18A2, TSHR, PRNP, DCTN1
Dysphoria	156 (32.64)	2 (16.67)	LEP, PRNP
Dizzy	135 (33.25)	19 (29.23)	TNXB
Fever	121 (16.78)	41 (29.08)	IL6, HLA-DRB1, PRNP, PRL, CRP
Thirst	99 (18.20)	1 (25.00)	LEP
Fatigue	79 (14.42)	49 (26.78)	HESX1, LHX3, TNXB, SLC18A2, TSHR, DNMT1, HLA-DRB1, PRNP, DCTN1, PROP1, TSHB, POU1F1
Blurred vision	69 (38.76)	19 (23.75)	HESX1, CLIP2, LIMK1, BAZ1B, PRNP, GTF2IRD1, GTF2I, RFC2, TBL2, ELN
Night sweats	53 (42.06)	38 (31.40)	SLC18A2, HLA-DRB1, DDC, PRNP, HMBS
Poor appetite	51 (11.26)	31 (36.90)	HMBS
Constipation	47 (13.35)	34 (24.46)	DDC, PRNP, RAI1, NR4A2, THRA, TSHB, POU1F1, FLII, HESX1, TSHR, THRB, HMBS, CLIP2, SNCAIP, LHX3, TRHR, TNXB, LIMK1, BAZ1B, GTF2IRD1, PROP1, RFC2, GTF2I, TBL2, ELN, CPOX
Amnesia	44 (66.67)	44 (32.35)	NPS, HCRT, IL6, HLA-DRB1, DNMT1, PRNP, HLA-DQB1, MOG, ZNF365
Tachypnea	33 (15.57)	20 (20.83)	HLA-DRB1, DCTN1
Emaciation	33 (23.91)	44 (29.73)	TSHR, HLA-DRB1, SLC9A6, PRNP, HLA-DQB1, SNCA, DCTN1, LEP
Loose stools	23 (8.68)	33 (31.43)	TSHR, NAGLU, DDC, HMBS, SGSH, CPOX, GNS
Headache	23 (9.54)	21 (32.31)	IL6
Rash	21 (10.66)	46 (26.29)	TNXB, IL6, HLA-DRB1, CRP, SIN3A
Body pain	19 (6.57)	36 (25.35)	CLIP2, TNXB, IL6, LIMK1, HLA-DRB1, BAZ1B, ELN, GTF2IRD1, GTF2I, HMBS, RFC2, TBL2, CPOX
Cough	16 (7.80)	28 (49.12)	HLA-DRB1
Consciousness disorder	16 (11.27)	28 (24.59)	IL6, TSHB

^a^The co-occurrences are presented as *n*/*N* (%), where *n* is the co-occurrence frequency of the symptom and insomnia in a textbook named *differential diagnosis of traditional Chinese medicine symptom*; *N* is the total occurrence frequency of symptom in this book. ^b^The overlap pathways are presented as *n*/*N* (%), where n is the number of overlapped enriched KEGG pathways between the symptom and insomnia; *N* is the total enriched KEGG pathways of the symptom.

Particularly, using hierarchical agglomerative clustering analysis (by the cluster map function in the Python Seaborn library)^62^, we identified 54 enriched pathways of 22 pathogenesis types and 5 main symptom clusters, such as (insomnia, fever, rash), (insomnia, body pain, emaciation, fatigue), (insomnia, loose stools, poor appetite), (insomnia, night sweats, headache), and (insomnia, constipation, emotional lability) for insomnia disorder (Fig. 6). For example, the overlapped pathways of insomnia-fever-rash cluster are involved in immune and infectious disease (e.g., herpes simplex infection). The related report that sleep–wake cycles have emerged as prominent regulators of the immune system and variations in sleep duration that occur in the natural setting have the potential to impact infectious disease risk^63^. The patient of insomnia-body pain-emaciation-fatigue cluster are associated with cancer^64,65^, and the related pathways include dysregulation of cancer transcriptional regulation. Other insomnia patients often show constipation and emotional lability after taking drugs^66^, and the pathways are related to the substance dependence, such as amphetamine addiction, alcoholism, and cocaine addiction.

**Fig. 7:**
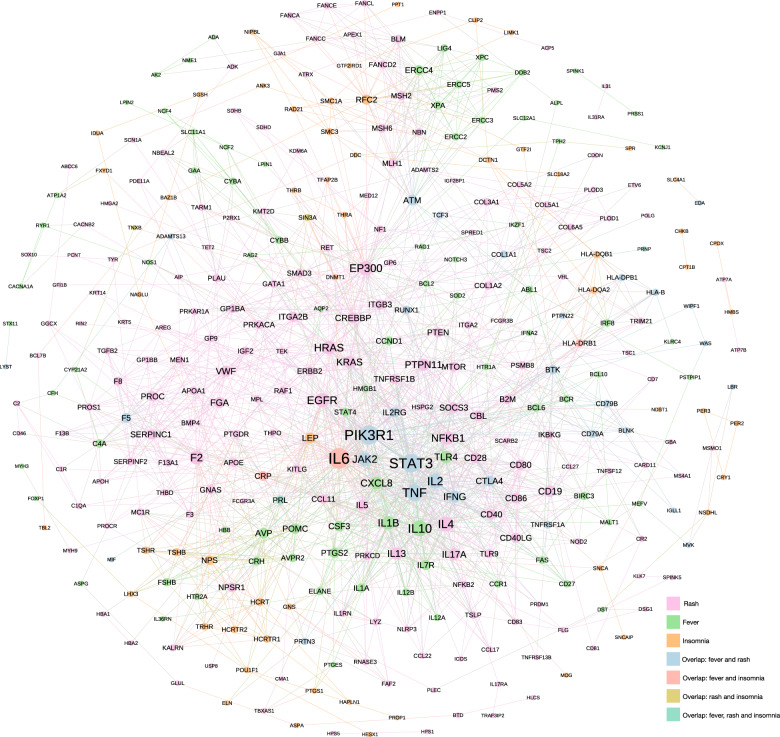
Construction the PPI network of insomnia-fever-rash cluster. We extracted a PPI subnetwork of insomnia-fever-rash symptom clusters which consisted of 363 nodes and 1860 edges. The nodes indicate the related genes of these symptoms in PPI network and edges represent the interactions of these genes in PPI network. Node size reflected the degree of symptom in the network (a high degree is represented by large node). Node colors represent genes associated with different symptoms.

In addition, we have extracted the PPI networks of the 5 insomnia-related symptom clusters (Fig. 7 and Supplementary Figs. 3–6) and obtained the enriched gene ontology terms of biological process (GO_BP) of the overlapping genes for each cluster (Table 4 and Supplementary Tables 3–5). We found that insomnia-fever-rash symptom cluster includes the cytokines (e.g., IL6, IL10, and IL1B) and inflammatory biomarkers (e.g., PIK3R1, STAT3, and TNF) as the hub genes in their associated PPI network and tends to be related to the inflammatory immune-related insomnia subtype involving the biological processes, such as B-cell differentiation, antigen processing and presentation, and cytokine-mediated signaling pathway (Fig. 7 and Table 4). We also found that genes in the network, such as PTGS2 and PTGS1, are targeted by a variety of nonsteroidal anti-inflammatory drugs (NSAIDs), including dexibuprofen, mefenamic acid, and bufexamac to improve symptoms of fever, rash, and insomnia^67–69^. It is similar and biomedical meaningful for the other 4 insomnia-related symptom clusters.

**Table 4:** The GO BP of overlapping genes enriched of insomnia-fever-rash cluster.

ID	GO_BP	*P*-value (*P* < 0.01)
1	Immune response	2.70E-12
2	Adaptive immune response	7.90E-07
3	B-cell receptor signaling pathway	5.80E-06
4	B-cell differentiation	1.30E-05
5	T-cell receptor signaling pathway	1.70E-05
6	Interferon-gamma-mediated signaling pathway	1.70E-05
7	Positive regulation of gene expression	2.10E-05
8	Positive regulation of nitric oxide biosynthetic process	1.10E-04
9	Platelet activation	1.20E-04
10	Growth hormone receptor signaling pathway	1.30E-04
11	Negative regulation of lipid storage	1.30E-04
12	Intrinsic apoptotic signaling pathway in response to DNA damage	1.50E-04
13	Cytokine-mediated signaling pathway	1.90E-04
14	Positive regulation of transcription from RNA polymerase II promoter	2.20E-04
15	Antigen processing and presentation	2.40E-04
16	Humoral immune response	2.70E-04
17	Negative regulation of apoptotic process	4.40E-04
18	JAK-STAT cascade involved in growth hormone signaling pathway	4.90E-04
19	T-cell costimulation	6.70E-04
20	Blood coagulation	6.90E-04
21	Defense response to protozoan	7.90E-04
22	Inflammatory response	1.40E-03
23	Positive regulation of sequence-specific DNA binding transcription factor activity	1.60E-03
24	Cellular response to lipopolysaccharide	2.00E-03
25	Tumor necrosis factor-mediated signaling pathway	2.20E-03
26	Positive regulation of NF-kappaB transcription factor activity	3.10E-03
27	Positive regulation of tyrosine phosphorylation of Stat3 protein	3.20E-03
28	Extrinsic apoptotic signaling pathway via death domain receptors	3.20E-03
29	Acute-phase response	3.30E-03
30	Positive regulation of B-cell proliferation	3.30E-03
31	Negative regulation of gene expression	3.40E-03
32	Extrinsic apoptotic signaling pathway	3.80E-03
33	Defense response to bacterium	4.00E-03
34	Viral process	4.10E-03
35	Positive regulation of vitamin D biosynthetic process	4.40E-03
36	Positive regulation of growth factor dependent skeletal muscle satellite cell proliferation	4.40E-03
37	Positive regulation of interferon-gamma production	4.60E-03
38	Positive regulation of tumor necrosis factor production	4.80E-03
39	Positive regulation of transcription, DNA-templated	5.20E-03
40	Neutrophil apoptotic process	6.60E-03
41	Positive regulation of calcidiol 1-monooxygenase activity	6.60E-03
42	Positive regulation of ERK1 and ERK2 cascade	6.70E-03
43	Positive regulation of T cell proliferation	7.70E-03
44	I-kappaB kinase/NF-kappaB signaling	7.70E-03
45	Regulation of cell proliferation	7.80E-03

To measure the function of overlapping genes in PPI network of insomnia-fever-rash cluster, we obtained the specific gene ontology function categories terms in biological process (GO_BP) of 38 overlapping genes (including the overlapping genes for two symptoms) for the cluster (*P*-value < 0.01).

## Discussion

Symptom phenotypes are the overt manifestations of disease observed by physicians and patients. However, most symptoms are non-specific and rarely identify a disease unambiguously. In fact, numerous diseases—including some of the most common ones such as cancer, cardiovascular disease, and HIV infection—may manifest unspecific symptoms (e.g., fatigue) in the early stage which often easily be ignored to regard as the asymptomatic phenomenon^5^. Therefore, it is a vital task to elucidate the underlying molecular mechanisms of symptoms, in particular the network mechanisms of them to investigate the pathogenesis of non-specificity of symptom phenotypes. However, the biological mechanisms of symptom phenotypes have rarely been addressed in systematic approach, which might largely be owing to the lack of high-quality symptom-gene associations data.

Here, we curated high-quality symptom-gene associations and quantitatively evaluated the network diversity of symptom phenotypes using a well-established network measure (i.e., node diversity). The results showed that the degree of un-specificity of symptoms could be represented by node diversity and we further found that the clinical diversity of symptom phenotypes could be partially explained by the molecular network diversity of symptom phenotypes (significant positive correlation between MGD and PD was detected; *PCC* = *0.49, P*-value = *2.14E-08)*. Furthermore, we evaluated the molecular diversity of diseases and found it is lower than those of symptom phenotypes. These results validate the advantages of disease diagnosis and the reliability of MGD for evaluating the diversity of symptom phenotypes. Overall, our work proposes a feasible approach to evaluate the diversity of symptom phenotypes and it could further be used for “symptom subtyping” as recent literature for establishing the new disease taxonomy^70^.

Particularly, as a recent hot research topic that has been intensively investigated in nursing science^71^. Various studies have identified significant symptom clusters (e.g., fatigue, depressive symptoms, and anxiety^72^) of the typical diseases during the nursing process, such as psychiatric diseases (e.g., depression and anxiety)^73^, cancer diseases (e.g., breast cancer, gastrointestinal cancer, lung cancer)^74^, and chronic diseases (e.g., chronic kidney disease, chronic obstructive pulmonary disease, type 2 diabetes)^75–77^. For example, related study found that patients with heart failure (HF) would manifest distinct symptom clusters, the weary (lack of energy, lack of appetite, and difficulty sleeping) and the dyspneic symptom clusters (shortness of breath, difficulty breathing when lying flat, and waking up breathless at night). Each one unit increase in mean distress score in the dyspneic symptom cluster doubled the risk for cardiac death and the risk of cardiac rehospitalization increased by 1.5 times for each one unit increase in mean distress score in the weary symptom cluster^78^. Therefore, it is a promising clinical analysis task to find significant symptom clusters involved in various disease conditions. It also emphasizes the importance of investigating and monitoring of symptom clusters which can help improve the capability of clinical diagnosis, treatment and predict the outcomes in patients rather than individual symptoms. Altogether, symptom clusters have proposed an effective approach for symptom subtyping, which would deliver population stratification with higher specificity than single symptom phenotype. In our study, using the molecular diversity measurement of symptom phenotypes, we further investigate the underlying network mechanisms of symptom clusters and why their clinical specificities could be obtained, which would finally be helpful to detect and understand various symptom subtypes involved in different disease conditions.

There still have several limitations for our work. First, the number of symptom-gene associations is limited, which is mainly owing to the focus of PGA on congenital hereditary diseases. In our study, most of the symptoms with gene associations belong to the nervous system, which would be result in certain deviations. However, the 341 symptoms in our work have covered 180 (46.63%) of symptoms in Medical Subject Heading vocabulary^67^ which ﻿was created and updated annually by the NLM since 1960s. This means that our results would deliver some kinds of reliable and useful knowledge for understanding the network mechanisms of the whole spectrum of symptom phenotypes. Second, the disparity of clinical and biomedical terminologies on symptom phenotypes is another obstacle to perform the translational medicine studies as our work. We found that clinical terminologies in clinical settings would tend to be in more specific granularities and the terms in biomedical data would be in higher levels. Therefore, the semantic mapping between different terminologies is a vital task for our study. This is further challenged by the cross-language translation difficulty involving Chinese and English languages. Actually, we have used the symptom cluster data in Chinese to construct the SCN, which would have the constraints of specific language (i.e., Chinese). In addition, the recordings of symptom clusters in Chinese and Chinese population would possibly influence the generalization of our results for other populations. Notwithstanding these plenty of challenges, we are convinced that advances in the field of symptom science will eventually enable us to substantially expand the data sources and thus promote the understanding of symptom phenotypes in the postgenomic era. In the future, we hope to identify novel and effective drug targets for symptom subtypes by incorporating the underlying network mechanisms of symptom diversity, so as to better serve the individualized diagnosis and treatment.

## Methods

### Basic datasets and preprocessing

We curated both clinical and molecular related data on symptom phenotypes to perform our study, which includes (i) clinical symptom manifestations from textbook, (ii) phenotype-genotype associations, (iii) protein interactome data, and (iiii) drug–targets associations.

#### Clinical symptom manifestations

We curated the data related to clinical symptoms derived from a well-recognized textbook named DDTS for clinicians in China, which contain 431 investigated symptoms and their symptom clusters (with 988 additional symptoms) in traditional Chinese medicine (TCM) clinical settings. This book is an important part of TCM syndrome differentiation and treatment, which reflects the use of TCM basic theory syndrome differentiation method for subtype analysis of symptoms. The characteristics of the same symptom in different clusters reflect the diversity and complexity of symptom in clinical settings. Therefore, the book could have served as a data source for exploring the diversity of symptoms.

#### Phenotype–genotype associations

We used an integrated PGA from DisGeNet^79^ and MalaCards^80^, which contains 110,407 associations with 11,362 unique diseases represented by UMLS CUI code and 13,271 unique genes.

#### Protein–protein interactions

The PPI were filtered from the human subset of STRING V11^23^ by the score threshold > =700, which include 17,185 distinct proteins and 420,534 high-quality interactions.

#### Drug–targets associations

The drug–targets associations obtained from the DrugBank database^30^, which is a comprehensive online database containing information on drugs and drug targets. Finally, we obtained 948 unique drugs and their 1451 targets for correlation analysis.

### Construction of symptom association network

In the DDTS, several established symptom clusters would be associated for each chief symptom. We considered symptom cluster as one record and constructed the SCN by symptom co-occurrence in symptom clusters and visualized by Gephi 0.9.2 software. To connect phenotypic and genetic data of symptoms in SCN, we manually mapped Chinese terms of symptoms in clinical data to English terms of symptoms in PGA by the trained medical researchers (e.g., Zixin Shu, Ning Xu, Chenxia Lu, Runshun Zhang) in our author list, thereby ensuring highly accurate terminological mappings. 252 (73.90%) English symptom terms with associated genes mapped to 116 Chinese symptom terms in SCN. Therefore, there is a phenomenon of multiple CUI code merging corresponding to one TCM symptom, for example, C0035021 and C0015967 were both mapped to发热 (i.e., fever). Finally, we obtained the genetic information of 116 symptoms in SCN by merging the genetic associations of the CUI code symptoms (Supplementary Table 1).

### Measuring the phenotypic diversity

We used node diversity^13^ to characterize the diversity of symptom phenotypes in the context of network, which have been successfully used for measuring disease diversity in recent studies^12,70^. The diversity *ϕ* of node *j* is based on the node bridging coefficient^81^ and defined by

$$\phi (j) = \mathop {\sum }\limits_{i\; \in N(i)} \frac{{\delta (i)}}{{k\left( i \right) - 1}}$$

where *k (i)* is the degree of node *i*, *N (i)* denotes its neighborhood, that is, the set of all its direct neighborhood and *δ (i)* is the total number of links leaving that neighborhood. The diversity *ϕ* is large for nodes with many neighbors that have out-going links themselves.

To evaluate the MD of phenotypes, we assume the molecular diversity of symptom phenotypes would largely lie on the related genes in the context of molecular network. For example, to quantify the MD (in terms of node diversity) of amnesia, we calculated all the node diversity values for the amnesia-related genes, such as MAPK1, EP300, and APP. Finally, we considered the MD of amnesia as 299.95 since we found that MAPK1 has the maximum node diversity of 299.95 among those genes. Furthermore, it is intuitively that node degree also could be considered as additional measure for molecular diversity.

### Shortest paths length between drug targets and symptom genes

Shortest paths are an important topological measurement for the analysis of social and biological networks^12^. Here, we utilize Dijkstra’s algorithm^82^ to find all shortest path lengths between drug targets and genes of symptom in the PPI network to help obtain 1-order drug targets and their related drugs for a given symptom phenotypes.

### Enrichment analysis

In order to identify molecular pathways and biological processes that could be impacted by the gene variations of each symptom cluster we used enrichment analysis. Pathway analysis offers the great power for discovering the biological functions underlying genes and proteins. The KEGG PATHWAY database is the main database in Kyoto Encyclopedia of Genes and Genomes (KEGG), and it consists of manually drawn reference pathway maps together with organism specific pathway maps^56^. Gene set enrichment analysis is a method of identifying classes of genes or proteins that are over-represented in a large set of genes or proteins and may be associated with disease phenotypes. We obtained the enriched KEGG pathways and gene ontology terms of biological process using the database for annotation, visualization, and integrated discovery (DAVID)^83^, which is a web-based online bioinformatics resource that aims to provide tools for the functional interpretation of large lists of genes/proteins.

### Reporting summary

Further information on research design is available in the Nature Research Reporting Summary linked to this article.

## Supplementary information

Supplementary Information

Reporting Summary

## Data Availability

All the relevant data supporting the findings of this study are included in the paper and its Supplementary material files.

## Code availability

The source codes and data are available at: https://github.com/shuzixin9212/symptom-diversity. The codes including the construction of the SCN and the calculation of node diversity. Node diversity algorithm was implemented using Java JDK 1.8. The other data analysis tasks were implemented using Python 3.7. In addition, we also provide two types of source files, (1) the clinical data used to construct SCN, the mapping data between Chinese and English terms of symptom phenotypes; (2) the symptom-gene associations and protein–protein interaction network data.
