# Supplementary material for: Diversity and molecular network patterns of symptom phenotypes
Source: NPJ Syst Biol Appl. 2021 Nov 30;7:41. doi: 10.1038/s41540-021-00206-5 (PMC8632989; doi:10.1038/s41540-021-00206-5)
Supplement: Supplementary file 1 — Supplementary Information [file 41540_2021_206_MOESM1_ESM.pdf]

Diversity and molecular network patterns of symptom phenotypes Supplementary File

In our study, the identification of symptom clusters can reduce the diversity of symptoms and improve specificity, which has been confirmed by the comparison of MD between symptom pairs and single symptoms. In addition, we have extracted the PPI networks of the insomnia related symptom clusters **(Supplementary Figure 2-5)** and obtained the enriched gene ontology terms of biological process (GO_BP) of the overlapping genes for each cluster **(Supplementary Table 3-5)**. These results show the unique molecular mechanism of each symptom cluster, which indicate that these insomnia symptom clusters typically describe several subtypes of insomnia disorder.

insomnia-body pain-emaciation-fatigue cluster includes the biomarkers, such as PIK3R1, PIK3CA and JAK2 as the hub genes in their associated PPI network and related to the cell-related metabolic process[1,2] (e.g. negative regulation of neuron apoptotic process) and cell differentiation and repair (e.g. nucleotide-excision repair). Targeting apoptosis has been developed as a common approach to effectively eliminate cancer cells, but it can cause common adverse reactions, such as insomnia and body pain[3]. In addition, we found HAPLN1 in the network was targeted by hyaluronic acid, which is a glycosaminoglycan used for the relief of joint pain, wound healing, ophthalmologic treatment and various other applications[4].

Insomnia-constipation-emotional lability cluster includes most constipation-related genes with higher degree in their associated PPI network, such as GCG, TAC1 and CASR, which formed a denser internal module characteristic with some of the genes for emotional lability (e.g. HTR2A and HTR2C) and insomnia (e.g. NPS and HCRT). HTR2A were targeted by various of psychotropic drugs, including minaprine and flupentixol[5,6]. The molecular mechanisms of the overlapped genes are involved hormone-mediated signaling pathways (e.g. steroid hormone, dopamine and peptide hormone), which critically affect behavioral adaptation to stress and are causally linked to emotional disorders[7].

Insomnia-loose stools-poor appetite cluster includes PIK3R1, PTPRC and CD19 with higher degree in their associated PPI network, CD19 is a target of chimeric antigen receptor T-cells used in the treatment of lymphoblastic leukemia, which with a combination of constitutional symptoms and signs of bone marrow failure, such as anemia, thrombocytopenia and leukopenia[8]. The function of the overlapped genes related to the hematogenic and immunoreactions process (e.g. B cell differentiation, protoporphyrinogen IX biosynthetic process and heme biosynthetic process). A lot of studies indicate that the presence of nutritional anemia was significantly associated with a higher likelihood of having insomnia[9]. We also found some targets (e.g. CNR1, CNR2 and CHRM1) in the network are related to gastrointestinal regulating drugs, including used for chemotherapy and postoperative drugs (e.g. dronabinol[10] and ramosetron[11]) and antidiarrheal agent (e.g. crofelemer[12] and furazolidone[13]).

Insomnia-night sweats-headache cluster includes some genes with higher degree related headache in the network, such as CTNNB1, TP53 and APP, are involved in the development of tumors[14]. The function of the overlapped genes related to the regulatory process of neurotransmitters (e.g. aminergic neurotransmitter loading into synaptic vesicle and positive regulation of nitric oxide biosynthetic process), whose imbalanced concentrations in the brain can cause symptoms such as headaches and insomnia[15,16].

Furthermore, we also provided other materials, including the correlation results of the disease molecular network diversity and related drug-targets diversity **(Supplementary Figure 1)**, the insomnia symptom cluster network results **(Supplementary Figure 2),** and related symptom data processing results **(Supplementary Table 1 and 2)**.

Supplementary CHECK LIST

Supplementary Table

1	A mapping list of 252 English symptom terms mapping into 116 Chinese terms	P. 3
2	A list of symptom terms filtered out with DP characteristic	P.11
3	The GO_BP of overlapping genes enriched of insomnia-body pain-emaciation-fatigue cluster	P.13
4	The GO_BP of overlapping genes enriched of insomnia-constipation-emotional lability cluster	P.16
5	The GO_BP of overlapping genes enriched of insomnia-loose stools-poor appetite cluster	P.17
6	The GO_BP of overlapping genes enriched of insomnia-night sweats-headache cluster	P.18

Supplementary Figure

1	Correlations of the disease molecular network diversity and related drug-targets diversity	P. 19
2	Construction of insomnia symptom cluster clinical association network	P. 20
3	Construction of insomnia-body pain-emaciation-fatigue cluster of PPI network	P. 21
4	Construction of insomnia-constipation-emotional lability cluster of PPI network	P. 22
5	Construction of insomnia-loose stools-poor appetite cluster of network	P.23
6	Construction of insomnia-night sweats-headache cluster of PPI network	P.24

Supplementary Table 1. A list of 252 CUI English symptom terms mapped into 116 symptom terms in SCN

Chinese terms in SCN	English terms in SCN	CUI code	CUI English terms from UMLS
发热	Fever	C0015967	Alteration in body temperature: hyperthermia, fever
		C0035021	Fever, famine
乏力	Fatigue	C0004093	Asthenia
		C0085632	Listlessness
		C0015672	Decreased energy
口渴	Thirst	C0085602	Polydypsia
情绪不稳	Emotional lability	C3887611	Restlessness marked
		C0086132	Depression symptom
		C0007398	Catatonic
		C0011570	Monopolar depression
		C0860609	Inappropriate crying
		C0085631	Abnormal excitement
烦躁	Dysphoria	C3887611	Restlessness marked
		C0085602	Polydypsia
纳差	Poor appetite	C0232462	Appetite decrease
		C0003123	Anorectic
头晕	Dizzy	C0012833	Dizzy
		C0007297	Car sickness
		C0001882	Air sickness
		C0026603	Motion sickness
		C0036494	Mal de mer
汗出	Sweating	C0700590	Diaphoresis excessive
		C0028081	Night sweat
便秘	Constipation	C0009806	Constipate
		C0401149	Constipation chronic
恶寒	Chill	C0085593	Chill
		C0021400	Influenza
不寐	Insomnia	C0851578	Disorder sleep
		C0037317	Sleep disturbance
		C0917801	Sleep disorder insomnia
		C0037316	Not enough sleeping
腹痛	Abdominal pain	C0000737	Abdomen pain
		C0262527	Intermittent abdominal pain
		C0016199	Pain flank
肢体痛	Body pain	C0004604	Pain back

		C0024031	Back pain lower back/aches, low back
		C0019559	Hip arthralgia
		C0151825	Ostalgia
		C0023222	Lower extremity pain musculoskeletal
		C0026858	Musculoskeletal pain
		C0231528	Muscle pain generalized
		C0030196	Limb pain
		C0003862	Pain joint
便溏	Loose stools	C0011991	Loose stools
头痛	Headache	C0018681	Headache, cephalalgia
呼吸急促	Tachypnea	C0020578	Hyperventilate
		C0013404	Respiratory difficulty
		C0476273	Distress respiratory
		C0231835	Respiration rate increased
咳嗽	Cough	C0010200	Cough
		C0019079	Bloody sputum
皮疹	Rash	C0013491	Ecchymoses
		C0015230	Exanthem
		C0015231	Exanthem subitum
		C0034150	Skin purpura
		C0031256	Petechia
		C0221263	Cafe au lait spot
		C0013595	Eczematous dermatitis
视力受损	Impaired vision	C0042798	Vision dim
		C0750903	Amblyopia strabismic
		C0344232	Blurred vision
		C0002418	Amblyopic
		C0271185	Metamorphopsia
视物模糊	Blurred vision	C0344232	Blurred vision
		C0002418	Amblyopic
		C0750903	Amblyopia strabismic
		C0042798	Vision dim
呕吐	Vomiting	C0027498	Nausea vomiting
		C0042963	Vomit
		C0221151	Vomit projectile
		C0020450	Hyperemesis gravidarum
恶心	Nausea	C0027498	Nausea vomiting
		C0027497	Queasy
意识障碍	Consciousness disorder	C0023380	Lethargy

		C0039070	Collapse fleeting
		C0011206	Delirium acute
		C0234428	Consciousness disturbance
		C0009421	Comatose
		C0041657	Consciousness loss
消瘦	Emaciation	C1262477	Weight loss
		C0041667	Low weight
盗汗	Night sweats	C0700590	Diaphoresis excessive
		C0028081	Night sweat
浮肿	Edema	C0013608	Cardiac edema
		C0151205	Periorbital edema
		C0424810	Periorbital swelling
		C0013604	Edematous
抽搐	Convulsion	C0751900	Motor tic
		C0009024	Clonus
		C0751495	Seizure focal
		C0026821	Cramp
		C0234166	Hyperekplexia
		C0014549	Seizure disorder tonic clonic
		C0027066	Myoclonic jerking
		C0037763	Spasm
		C2169806	Tic
		C0036572	Convulsion
昏迷	Coma	C0041657	Consciousness loss
		C0009421	Comatose
皮肤斑块	Skin patches	C0034150	Skin purpura
		C0031256	Petechia
		C0013491	Ecchymoses
		C0221263	Cafe au lait spot
皮肤瘙痒	Skin pruritus	C0033774	Skin pruritus
尿少	Oliguria	C0028961	Urine output decreased
		C0003460	Anurias
腹泻	Diarrhea	C0239181	Diarrhea intermittent
		C0740441	Diarrhea acute
腰痛	Low back pain	C0024031	Back pain lower back/aches, low back
		C0016199	Pain flank
		C0158252	Degenerative disc disease
		C0021818	Intervertebral disc disease
胸痛	Chest pain	C0008031	Pain chest

		C0340288	Angina stable
		C0002963	Angina variant <prinzmetal>
		C0002962	Angina
		C0002965	Crescendo angina
关节痛	Joint pain	C0019559	Hip arthralgia
		C0003862	Pain joint
尿不利	Difficulty in urination	C0028961	Urine output decreased
鼻塞	Stuffy nose	C0027424	Congestion nasal
健忘	Amnesia	C0338591	Global transient amnesia
		C0751295	Memory loss or impairment
		C0236795	Amnesia dissociative
		C0233795	Amnesia anterograde
		C0002622	Amnesias
		C0542476	Forgetful
		C0002624	Amnesia retrograde
下肢无力	Lower extremity weakness	C0577655	Quadricep weakness
		C1836296	Lower extremity weakness
		C0221166	Paraparesis
气喘	Asthma	C0677600	Inhalatory stridor
		C0038450	Stridors
嗳气	Belching	C0019521	Hiccoughs
目痛	Eye pain	C0151827	Pain eye
呼吸困难	Dyspnea	C0476273	Distress respiratory
		C0231807	Dyspnea exertional
		C1145670	Failure respiratory
		C0013405	Dyspnea, paroxysmal
		C0013404	Respiratory difficulty
便血	Hemafecia	C0474585	Black color stools
		C0017181	Gastrointestinal bleed
四肢痛	Limb pain	C0030196	Limb pain
		C0003862	Pain joint
		C0019559	Hip arthralgia
		C0023222	Lower extremity pain musculoskeletal
鼻衄	Epistaxis	C3809715	Epistaxis recurrent
抑郁	Depression	C0086132	Depression symptom
		C0178417	Anhedonia
		C3178803	Anhedonia, social
		C0011570	Monopolar depression
耳聋	Deafness	C1691779	Hearing disorder, cochlear

		C0018780	Frequencies hearing high loss
		C0392704	Cortical deafness
		C0018777	Deafness, conductive
		C0004310	Auditory disorder process
		C0018784	Deafness sensorineural
		C0018781	Hearing disorder, noise-induced
		C0155552	Hearing loss mixed
		C0018776	Deafness, central
		C1384666	Decreased hearing
		C0033074	Presbycusis
		C0018775	Hearing loss bilateral
颤抖	Tremor	C0040822	Tremors
		C0234376	Tremor action
		C0231531	Fibrillation muscle
		C1608410	Head titubation
		C0476217	Abnormal head movement
		C0234378	Postural tremor
		C0015644	Fasciculation
皮肤干燥	Dry skin	C0151908	Dry skin
畏光	Photophobia	C4020887	Photodysphoria
		C0085636	Light sensitivity
活动不利	Difficulty in movement	C0022408	Disorder joint
		C0233565	Bradykinesia
		C0752210	Paroxysmal dyskinesia
		C0152031	Joint swollen
		C0162298	Stiffness joints
月经过多	Menorrhagia	C0232943	Metromenorrhagia
尿失禁	Urine incontinence	C0042024	Urine incontinence
阳痿	Impotence	C1961100	Erectile dysfunction adverse event
瘫痪	Paralysis	C0030486	Extremity paralysis, lower
		C0030552	Paralysis partial
		C0037771	Paraparesis spastic
		C0018989	Paresis of one side of body
		C0018991	Paralysis one side of body/hemiplegia
		C0270790	Quadriparesis
尿痛	Painful urination	C0013428	Painful urination
发少	Thin hair	C1860844	Sparse, thin hair
口疮	Aphtha	C0149745	Ulcer mouth
		C0853945	Oral mucosa blister

下肢痛	Lower limb pain	C0023222	Lower extremity pain musculoskeletal
嗜睡	Lethargy	C0917799	Sleeping excessive
		C0694563	Eds/excessive daytime sleepiness
		C0023380	Lethargy
关节不利	Disorder joint	C0152031	Joint swollen
		C0022408	Disorder joint
		C0162298	Stiffness joints
口噤	Jaw spasm	C0041105	Jaw spasm
呕血	Hematemesis	C0018926	Emesis bloody
肥胖	Obesity	C2362324	Pediatric obesity
		C0028754	Obese
		C0028756	Obesity extreme
		C0497406	Over weight
口眼歪斜	Facial paralysis	C0015469	Facial paralysis
半身不遂	Hemiplegia	C0018991	Paralysis one side of body/hemiplegia
		C0018989	Paresis of one side of body
言语不利	Speech scanning	C0349391	Apraxia verbal
		C3495144	Apraxia, articulatory
		C0023015	Language handicap
		C0282513	Primary progressive aphasia
		C0917814	Aphasia expressive
		C0002018	Alexia
		C0476254	Dyslexia
		C0003910	Articulation disorder
		C0973461	Dysphasia
		C0003537	Alogia
		C0751706	Aphasia, progressive nonfluent
		C0264611	Apraxia of speech
		C0003113	Anomia
		C0278184	Speech scanning
		C0003550	Broca aphasia
咳血	Bloody sputum	C0019079	Bloody sputum
牙痛	Dental pain	C0040460	Dental pain
胃脘嘈杂	Brash	C0018834	Brash
声哑	Voice hoarseness	C0019825	Voice hoarseness
		C0241700	Voice fatigue
角弓反张	Opisthotonos	C0151818	Opisthotonos
黄疸	Jaundice	C0022346	Yellow skin
		C0022354	Jaundice obstructive

尿血	Hemoglobinuria	C0019048	Hemoglobinuria
智力低下	Dyscalculia	C0869474	Dyscalculia
		C3714756	Disabilities intellectual
		C0020796	Profoundly mentally retarded
		C0917816	Deficiency mental
肢体强直	Rigidity	C0026837	Rigidity
		C0027125	Myotonias
		C0026826	High muscle tone
		C0026838	Spasticity muscle
		C0751359	Myotonia, percussion
		C0151564	Cog wheel rigidity
肢体痿弱	Body weakness	C0746674	Muscle weakness generalized
		C0270948	Neurogenic muscular atrophy
		C0026846	Atrophy muscle
		C0151786	Weakness muscle
		C0872084	Sarcopenia
嗅觉减退	Hyposmia	C0003126	Smell loss
		C2364082	Hyposmia
里急后重	Tenesmus	C0232726	Tenesmus, rectal
听力减退	Decreased hearing	C1384666	Decreased hearing
背痛	Pain Back	C0004604	Pain back
喜食异物	Pica	C0240928	Salt craving
		C0031873	Pica
鼻翼煽动	Nasal Flaring	C0277873	Nasal flaring
发育迟缓	Failure to thrive	C0234861	Chat cri du
		C0015544	Failure to thrive syndrome
		C3552463	Very poor growth
多食	Excessive Eating	C0020505	Excessive eating
厌食	Anorexia	C0003123	Anorectic
手舞足蹈	Chorea	C0699731	Hereditary chorea
		C0393584	Benign hereditary chorea
		C0152113	Sydenhams chorea
		C0008489	Chorea disorder
眩晕	Vertigo	C0155502	Vertigo benign positional
		C0042571	Vertigo subjective
瞳神缩小	Pupillary constriction	C0026205	Pupillary constriction
疔肿	Boil	C0242301	Boil
目偏视	Amblyopia strabismic	C0750903	Amblyopia strabismic
手足蠕动	Athetoid movement	C0004158	Athetoid movement

视物变形	Metamorphopsia	C0271185	Metamorphopsia
失明	Blindness	C0155320	Blindness cortical
		C0456909	Blind
妄听	Hallucinations auditory	C0233762	Hallucinations auditory
头震颤	Head tremor	C0476217	Abnormal head movement
		C1608410	Head titubation
目浮肿	Periorbital edema	C0151205	Periorbital edema
		C0424810	Periorbital swelling
小便不通	Anurias	C0003460	Anurias
不耐久立	Orthostatic intolerance	C1535893	Orthostatic intolerance
		C0241237	Standing difficulty
多尿	High urine output	C0032617	High urine output
言语迟钝	Delay language	C0750927	Apraxia, developmental verbal
		C0236828	Developmental articulation disorder
		C0023012	Delay language
		C0023014	Developmental disorder language
		C0241210	Speaking delay
精神失常	Subacute confusional state Nos	C0154333	Subacute confusional state nos
小便困难	Anurias	C0003460	Anurias
关节肿胀	Joint swollen	C0152031	Joint swollen
幻觉	Hallucination	C0235153	Sensory hallucination
		C0233762	Hallucinations auditory
		C0233763	Hallucinations visual
		C0233773	Hallucinations hypnogogic
昏睡	Lethargy	C0023380	Lethargy
关节僵硬	Stiffness joints	C0162298	Stiffness joints
颌下痛	Jaw bone pain	C0236000	Jaw bone pain
足癣	Tinea pedis	C0040259	Tinea pedis
不能出声	Absence of voice	C0003564	Absence of voice
爪甲薄	Nail thinness	C0423823	Nail thinness
下肢僵硬	Leg muscle stiffness	C4024610	Leg muscle stiffness
鸭行步态	Gait waddling	C0231712	Gait waddling
内收足	In toe	C0231791	In toe
目凸	Ocular proptosis	C0015300	Ocular proptosis

We mapped English terms with genetic information into Chinese terms through manual processing to ensure the accuracy of results. 252 (73.90%) English symptom terms with associated genes mapped to 116 Chinese symptom terms in SCN. Finally, we obtained the genetic information of 116 symptoms in SCN by merging the genetic associations of the CUI code symptoms. For example, Genes related to the fever include the genes of C0035021 and C0015967 because of they all mapped to fever symptom.

Supplementary Table 2. A list of symptom terms filtered out with DP characteristic

CUI code	Symptom English name	CUI code	Symptom English name
C0001925	Albuminuria	C0032914	Toxemia
C0002103	Rhinitis, atopic	C0033377	Caudal displacement
C0003615	Appendicitis	C0033687	Proteinurias
C0005612	Infant birthweight	C0033790	Pseudobulbar palsy
C0005697	Neurogenic bladder	C0033922	Psychomotor disorder
C0005779	Clotting	C0033931	Psychophysiological dysfunction
C0005904	Alteration in body temperature	C0034063	Edema lung
C0005910	Weight	C0034124	Pupillary disorder
C0006271	Inflammation of the bronchioles	C0034151	Hyperglobulinemic purpura
C0006277	Bronchitis	C0034152	Henoch schonlein syndrome
C0006285	Bronchial pneumonia	C0034155	Thrombotic thrombocytopenic purpura
C0007642	Cellulitis nos	C0034933	Abnormal reflexes
C0007758	Cerebellar ataxia	C0035078	Failure kidney
C0007815	Cerebrospinal fluid rhinorrhoea	C0035229	Respiratory function impaired
C0007939	Syphilis chancre	C0035232	Diaphragmatic paralysis
C0013395	Indigestion	C0035455	Rhinitis
C0014118	Inflammation of the heart valve	C0036980	Cardiocirculatory collapse
C0014335	Enteritides	C0037199	Sinus infection
C0015930	Syndrome fetal distress	C0038868	Supranuclear palsy progressive
C0017494	Angular gyrus syndrome	C0039621	Tetani
C0019080	Bleed_nos problem	C0040034	Thrombocytopenia
C0019214	Hepatosplenomegaly	C0040416	Pupil adie
C0019937	Cervical sympathetic paralysis syndrome	C0040584	Tracheitis
C0020438	Hypercalciuria	C0041976	Inflammation of urethra
C0020440	Co2 retention	C0042384	Vasculitis, nonspecific
C0020455	Hypergammaglobulinemia	C0043068	Acute fulminant meningococcal bacteremia
C0020538	Hbp	C0043094	Weight gain
C0020615	Hypoglycemia nos	C0043352	Absent salivary secretion
C0020641	Hypopyon	C0079748	Lymphoma lymphoblastic
C0020672	Body temperature decreased	C0085128	Cardiac output elevated
C0021359	Infertility	C0085642	Asphyxia reticularis
C0021368	Inflammation	C0085650	Purpura fulminans
C0022116	Ischemia	C0086437	Joint hypermobility
C0022541	Kearn sayer syndrome	C0086523	Laryngeal paralysis
C0022638	Ketosis	C0086565	Liver function abnormal
C0022660	Acute kidney failure	C0086666	Preinfarction syndrome
C0023530	Leukopenia	C0151311	Cranial nerve palsy
C0023533	Vaginal discharge white	C0151740	Intracranial hypertension
C0024282	Lymphocytosis	C0152020	Atony stomach

C0024312	Lymphocytopenia	C0152227	Tearing excessive
C0025287	Meningitis-Like/dupre's syndrome	C0152459	Striae
C0025309	Meningoencephalitis	C0155765	Capillaries
C0027796	Neuralgias	C0158157	Joint effusion of other specified site
C0028734	Nocturnal frequency	C0162429	Dietary deficiency
C0029089	Muscle paralysis eye	C0162674	Chronic progressive paralysis of the external eye muscle
C0030201	Pain, postoperative	C0206146	Myocardial stunning
C0031154	Peritonitis,nos	C0206160	Retic count elevated
C0032285	Pneum	C0227791	Discharge vaginal
C0032768	Neuralgia postherpetic	C0231616	Beevor sign
C0231678	Ulnar deviation of the wrists	C0438434	Ring scotoma
C0234132	Pyramidal sign	C0525041	Cognitive symptoms
C0234133	Extrapyramidal symptom	C0684219	Fibrillary chorea
C0234979	Dysdiadochokinesia	C0686347	Dyskinesia tardive
C0240735	Personality change	C0700078	Deep tendon reflex decrease
C0240991	Sensory ataxia	C0740651	Abdomen symptom
C0242644	Brown sequard syndrome	C0751401	Ophthalmoparesis
C0242700	Adaptation syndrome, space	C0752252	Neuromuscular manifestation
C0242706	Hyperoxia	C0857305	Thrombocytopenia purpura
C0271097	Graefe-Usher syndrome	C0878773	Bladder hyperactive
C0271215	Blindness legal	C0936244	Developmental gerstmann syndrome
C0272412	Spleen abscess	C1268935	Congenital thrombotic thrombocytopenic purpura
C0311389	Nonspecific urethritis	C1403891	Adrenal hemorrhage syndrome
C0332573	Macula	C1568248	Usher Syndrome, Type II
C0332606	Facies elfin	C1568249	Usher Syndrome, Type II
C0349464	Wernicke-korsakoff psychosis	C1838579	Pseudobulbar signs
C0393588	Dystonias paroxysmal	C1838869	Proximal neurogenic muscle weakness
C0393756	Alcohol hangover	C1850830	Exercise-induced muscle pain
C0398650	Idiopathic thrombocytopenia purpura	C1868703	Bacterial tracheitis
C0427190	Ataxia, truncal	C1956258	Familial thrombotic thrombocytopenic purpura
C1963137	Hydrocephalus adverse event	C3203358	Alveolar hypoventilation
C1963154	Renal failure adverse event	C3668816	Inflammation of non-human mammary gland
C2242577	Oromandibular dystonia	C3854173	Pre-renal acute kidney injury
C2931205	Usher syndrome, Type I	C4021567	Central heterochromia
C2936821	Spinal cerebrospinal fluid leak	C4025720	Pseudobulbar behavioral symptoms
C4042891	Sleep wake disorders		

We manually reviewed and removed 149 symptoms without clear meaning under the guidance of medical and language experts to ensure the accuracy of results.

Supplementary Table 3. The GO_BP of overlapping genes enriched of insomnia-body pain-emaciation-fatigue cluster

ID	GO_BP	P-Value (P<0.01)
1	nucleotide-excision repair, DNA incision	1.30E-09
2	mismatch repair	3.30E-08
3	nucleotide-excision repair, DNA incision, 5'-to lesion	4.60E-08
4	nucleotide-excision repair, preincision complex stabilization	8.40E-08
5	nucleotide-excision repair	8.80E-08
6	response to UV-B	1.00E-07
7	nucleotide-excision repair, DNA incision, 3'-to lesion	1.40E-07
8	nucleotide-excision repair, preincision complex assembly	4.70E-07
9	DNA repair	6.30E-07
10	global genome nucleotide-excision repair	7.90E-07
11	defense response to protozoan	3.10E-06
12	response to lipopolysaccharide	3.20E-06
13	nucleotide-excision repair, DNA duplex unwinding	5.70E-06
14	response to drug	6.30E-06
15	negative regulation of apoptotic process	6.90E-06
16	immune response	1.90E-05
17	regulation of apoptotic process	2.20E-05
18	negative regulation of interleukin-17 production	2.50E-05
19	response to glucocorticoid	2.80E-05
20	B cell differentiation	3.00E-05
21	UV protection	3.40E-05
22	aging	3.40E-05
23	inflammatory response	4.20E-05
24	determination of adult lifespan	4.40E-05
25	positive regulation of ERK1 and ERK2 cascade	4.90E-05
26	transcription-coupled nucleotide-excision repair	5.30E-05
27	positive regulation of tyrosine phosphorylation of Stat3 protein	5.40E-05
28	response to UV	8.10E-05
29	cytokine-mediated signaling pathway	8.30E-05
30	somatic hypermutation of immunoglobulin genes	8.50E-05
31	negative regulation of neuron apoptotic process	8.60E-05
32	positive regulation of interferon-gamma production	1.20E-04
33	response to iron ion	1.20E-04
34	intrinsic apoptotic signaling pathway in response to DNA damage	1.30E-04
35	T cell receptor signaling pathway	1.60E-04
36	antigen processing and presentation	2.30E-04
37	positive regulation of apoptotic process	2.40E-04
38	apoptotic process	2.70E-04

39	positive regulation of T cell proliferation	3.30E-04
40	regulation of T cell receptor signaling pathway	4.40E-04
41	regulation of mitotic cell cycle phase transition	4.40E-04
42	response to estrogen	4.40E-04
43	negative regulation of interferon-gamma production	4.70E-04
44	reciprocal meiotic recombination	5.80E-04
45	positive regulation of gene expression	5.90E-04
46	positive regulation of lymphocyte proliferation	6.10E-04
47	interferon-gamma-mediated signaling pathway	6.20E-04
48	T cell costimulation	8.80E-04
49	cellular response to DNA damage stimulus	9.90E-04
50	positive regulation of cell proliferation	1.00E-03
51	response to molecule of bacterial origin	1.00E-03
52	macrophage activation	1.30E-03
53	response to auditory stimulus	1.30E-03
54	negative regulation of neuron death	1.40E-03
55	UV-damage excision repair	1.60E-03
56	T-helper 1 type immune response	1.90E-03
57	activation of protein kinase activity	1.90E-03
58	positive regulation of tumor necrosis factor production	2.20E-03
59	regulation of gene expression	2.20E-03
60	positive regulation of T cell mediated cytotoxicity	2.20E-03
61	response to magnesium ion	2.20E-03
62	innate immune response	2.50E-03
63	response to hypoxia	2.60E-03
64	negative regulation of B cell proliferation	2.60E-03
65	positive regulation of transcription from RNA polymerase II promoter	2.60E-03
66	response to copper ion	3.00E-03
67	isotype switching	3.00E-03
68	negative regulation of growth of symbiont in host	3.40E-03
69	defense response to Gram-negative bacterium	3.40E-03
70	cellular response to lipopolysaccharide	3.40E-03
71	protein phosphorylation	3.50E-03
72	humoral immune response	3.80E-03
73	positive regulation of tyrosine phosphorylation of Stat5 protein	3.80E-03
74	antigen processing and presentation of peptide or polysaccharide antigen via MHC class II	3.80E-03
75	positive regulation of T cell activation	4.30E-03
76	positive regulation of osteoclast differentiation	4.80E-03
77	muscle cell cellular homeostasis	4.80E-03
78	regulation of inflammatory response	5.00E-03
79	negative regulation of transcription from RNA polymerase II promoter	6.00E-03
80	positive regulation of NF-kappaB transcription factor activity	6.10E-03

81	positive regulation of JAK-STAT cascade	6.40E-03
82	positive regulation of peptidyl-serine phosphorylation	6.70E-03
83	negative regulation of gene expression	6.80E-03
84	nucleotide-excision repair, DNA damage recognition	7.00E-03
85	positive regulation of transcription, DNA-templated	7.30E-03
86	post-embryonic development	7.50E-03
87	cell cycle arrest	7.50E-03
88	response to interferon-gamma	7.60E-03
89	wound healing	9.60E-03

To measure the function of overlapping genes in PPI network of insomnia-body pain-emaciation-fatigue cluster, we obtained the specific gene ontology function categories terms in biological process (GO_BP) of 94 overlapping genes (including the overlapping genes for two symptoms) for the cluster (*P value<0.01)*.

Supplementary Table 4. The GO_BP of overlapping genes enriched of insomnia-constipation-emotional lability cluster

ID	GO_BP	P-Value (P<0.05)
1	hormone-mediated signaling pathway	9.90E-04
2	intracellular receptor signaling pathway	1.80E-03
3	transcription initiation from RNA polymerase II promoter	2.00E-03
4	transition between slow and fast fiber	3.30E-03
5	female courtship behavior	3.30E-03
6	steroid hormone mediated signaling pathway	4.00E-03
7	positive regulation of transcription from RNA polymerase II promoter	4.90E-03
8	serotonin transport	5.00E-03
9	aminergic neurotransmitter loading into synaptic vesicle	6.70E-03
10	Type I pneumocyte differentiation	8.30E-03
11	cell-cell signaling	8.50E-03
12	organ morphogenesis	1.00E-02
13	nervous system development	1.20E-02
14	monoamine transport	1.30E-02
15	protoporphyrinogen IX biosynthetic process	1.50E-02
16	dopamine biosynthetic process	1.70E-02
17	peptide hormone processing	2.60E-02
18	heme biosynthetic process	3.40E-02
19	regulation of transcription, DNA-templated	3.50E-02
20	dopaminergic neuron differentiation	3.60E-02
21	transcription, DNA-templated	3.80E-02

To measure the function of overlapping genes in PPI network of insomnia-constipation-emotional lability cluster, we obtained the specific gene ontology function categories terms in biological process (GO_BP) of 30 overlapping genes (including the overlapping genes for two symptoms) for the cluster (*P value<0.05)*.

Supplementary Table 5. The GO_BP of overlapping genes enriched of insomnia-loose stools-poor appetite cluster

ID	GO_BP	P-Value (P<0.05)
1	glycosaminoglycan catabolic process	1.50E-04
2	protoporphyrinogen IX biosynthetic process	6.40E-03
3	cell-cell signaling	1.40E-02
4	heme biosynthetic process	1.50E-02
5	immune response	3.50E-02
6	type I interferon signaling pathway	4.50E-02
7	B cell differentiation	4.60E-02

To measure the function of overlapping genes in PPI network of insomnia-loose stools-poor appetite cluster, we obtained the specific gene ontology function categories terms in biological process (GO_BP) of only 13 overlapping genes (including the overlapping genes for two symptoms) for the cluster (*P value<0.05)*.

Supplementary Table 6. The GO TERM of overlapping genes enriched insomnia-night sweats-headache cluster

ID	GO TERM_BP	P-Value (P<0.05)
1	negative regulation of cell proliferation	1.90E-03
2	thrombopoietin-mediated signaling pathway	2.00E-03
3	aminergic neurotransmitter loading into synaptic vesicle	2.60E-03
4	positive regulation of protein import into nucleus, translocation	6.50E-03
5	negative regulation of interferon-gamma production	1.80E-02
6	response to antibiotic	2.10E-02
7	positive regulation of cell differentiation	2.40E-02
8	positive regulation of tyrosine phosphorylation of Stat3 protein	2.50E-02
9	positive regulation of nitric oxide biosynthetic process	2.80E-02
10	negative regulation of apoptotic process	3.40E-02
11	negative regulation of sequence-specific DNA binding transcription factor activity	3.90E-02
12	negative regulation of protein phosphorylation	3.90E-02
13	cellular response to drug	4.40E-02

To measure the function of overlapping genes in PPI network of insomnia-night sweats-headche cluster, we obtained the specific gene ontology function categories terms in biological process (GO TERM_BP) of only 12 overlapping genes (including the overlapping genes for two symptoms) for the cluster (*P value<0.05)*.

Supplementary Figure 1. Correlations of the disease molecular network diversity and related drug-targets diversity

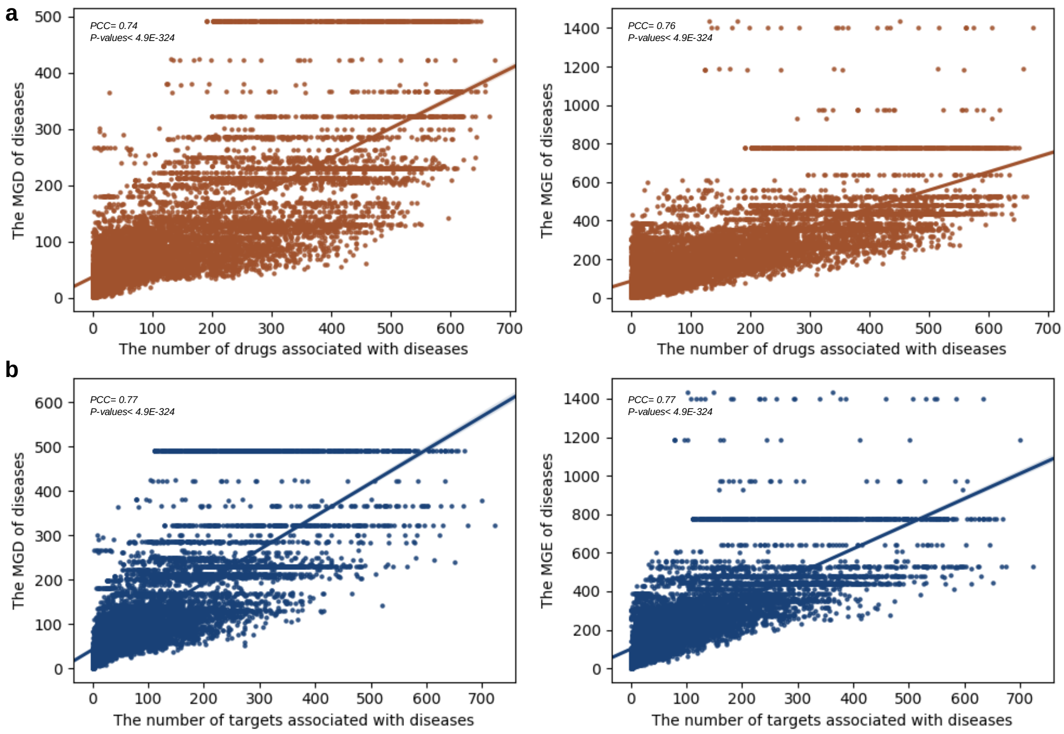

a. Correlations between the molecular network diversity of diseases and the number of related drugs. b. Correlations between the molecular network diversity of diseases and the number of related drug-targets. Here, 12563 diseases with gene associations were extracted from Malacards for the analysis of molecular network diversity. These results indicate that diseases with higher diversity in the molecular network may tend to have higher number of drug targets. This is similar for molecular network diversity of symptoms.

Supplementary Figure 2. Construction of insomnia symptom cluster clinical association network

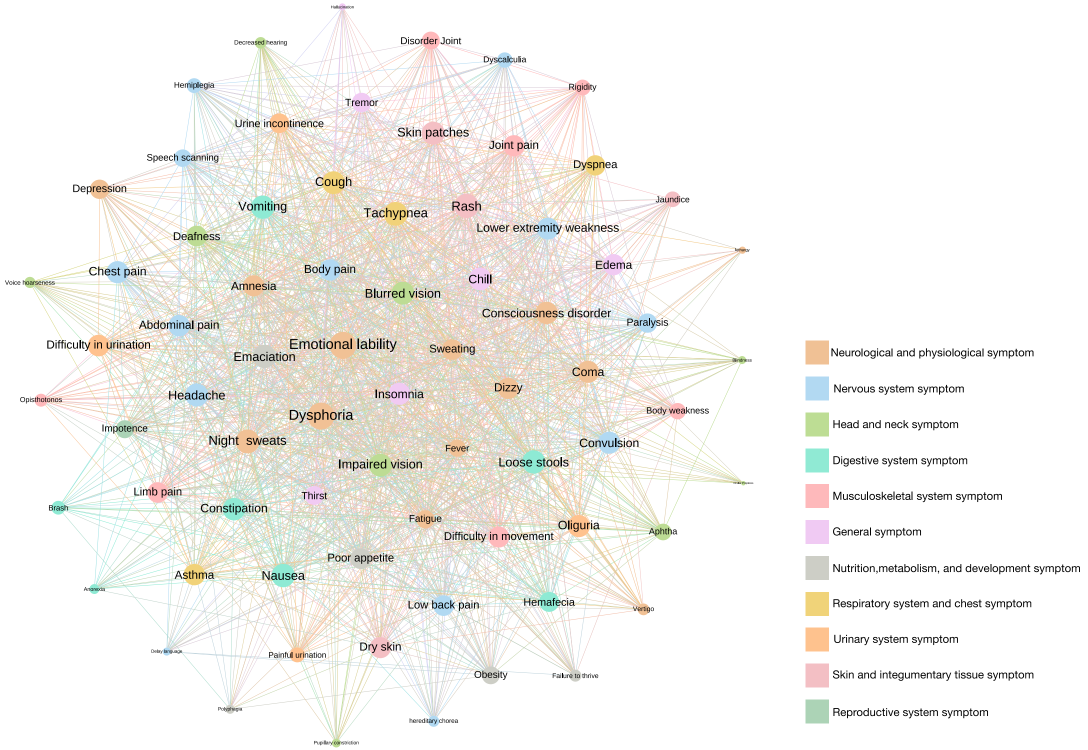

We extracted a subnetwork of insomnia-related symptom clusters from SCN which consisted of 73 nodes and 1636 edges. The nodes represent the symptoms (including insomnia and 72 symptoms in insomnia clusters) and the edges represent the clinical co-occurrences of them in SCN. Node size reflects the diversity of symptom phenotype in SCN (a high diversity is represented by large node). Node color reflects the corresponding system class according to the Symptom Ontology which was adopted as a standard ontology by the BioPortal. A total of 11 systems are involved in insomnia related symptoms, which 36.2% of symptoms related to neurological and physiological systems, such as abdominal pain, amnesia and dysphoria.

Supplementary Figure 3. Construction of insomnia-body pain-emaciation-fatigue cluster of PPI network

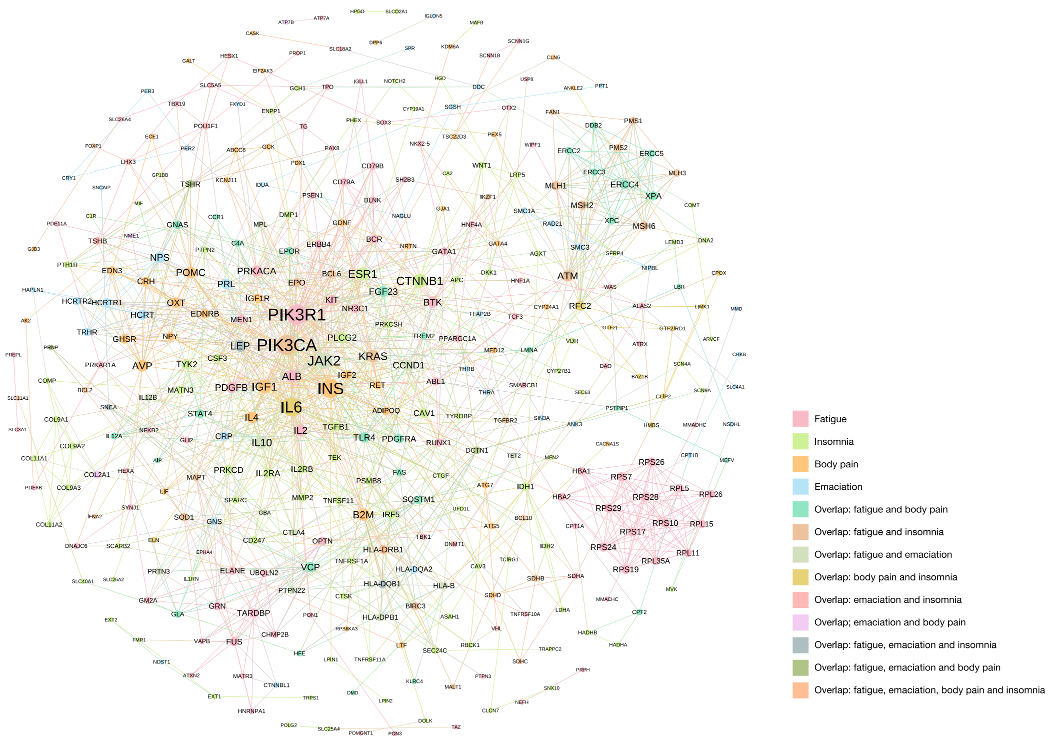

We extracted a PPI subnetwork of insomnia-body pain-emaciation-fatigue symptom clusters which consisted of 362 nodes and 1385 edges. The nodes represent the related genes of these symptoms in PPI network and edges represent the interactions of these genes in PPI network. Node size reflects the degree of symptom in the network (a high degree is represented by large node). Node color represents genes associated with different symptoms. PIK3R1, PIK3CA and JAK2 as the hub genes in their associated PPI network and related to the cell-related metabolic process[1,2] (e.g. negative regulation of neuron apoptotic process) and cell differentiation and repair (e.g. nucleotide-excision repair). In addition, we found HAPLN1 in the network was targeted by hyaluronic acid, which is a glycosaminoglycan used for the relief of joint pain, wound healing, ophthalmologic treatment and various other applications[4].

Supplementary Figure 4. Construction of insomnia-constipation-emotional lability cluster of PPI network

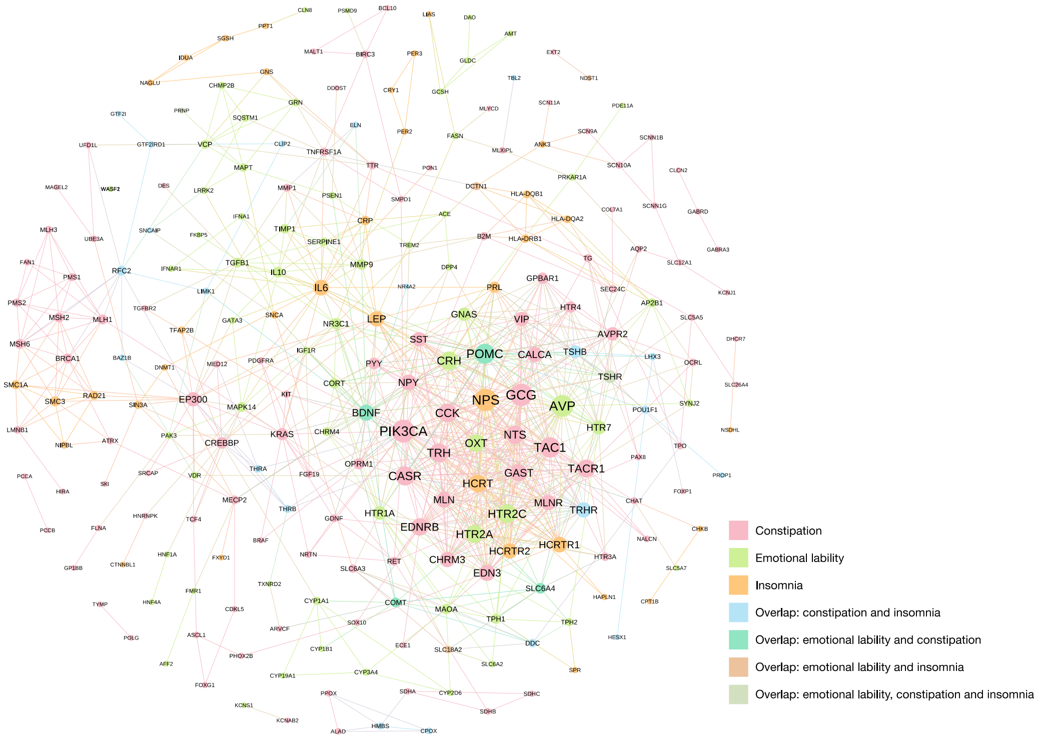

We extracted a PPI subnetwork of insomnia-constipation-emotion lability symptom clusters which consisted of 241 nodes and 932 edges. The nodes represent the related genes of these symptoms in PPI network and edges represent the interactions of these genes in PPI network. Node size reflects the degree of symptom in the network (a high degree is represented by large node). Node color represents genes associated with different symptoms. Most constipation-related genes with higher degree in their associated PPI network, such as GCG, TAC1 and CASR, which formed a denser internal module characteristic with some of the genes for emotional lability (e.g. HTR2A and HTR2C) and insomnia (e.g. NPS and HCRT). HTR2A were targeted by various of psychotropic drugs, including minaprine and flupentixol[5,6]. The molecular mechanisms of the overlapped genes are involved hormone-mediated signaling pathways (e.g. steroid hormone, dopamine and peptide hormone), which critically affect behavioral adaptation to stress and are causally linked to emotional disorders[7].

Supplementary Figure 5. Construction of insomnia-loose stools-poor appetite cluster of network

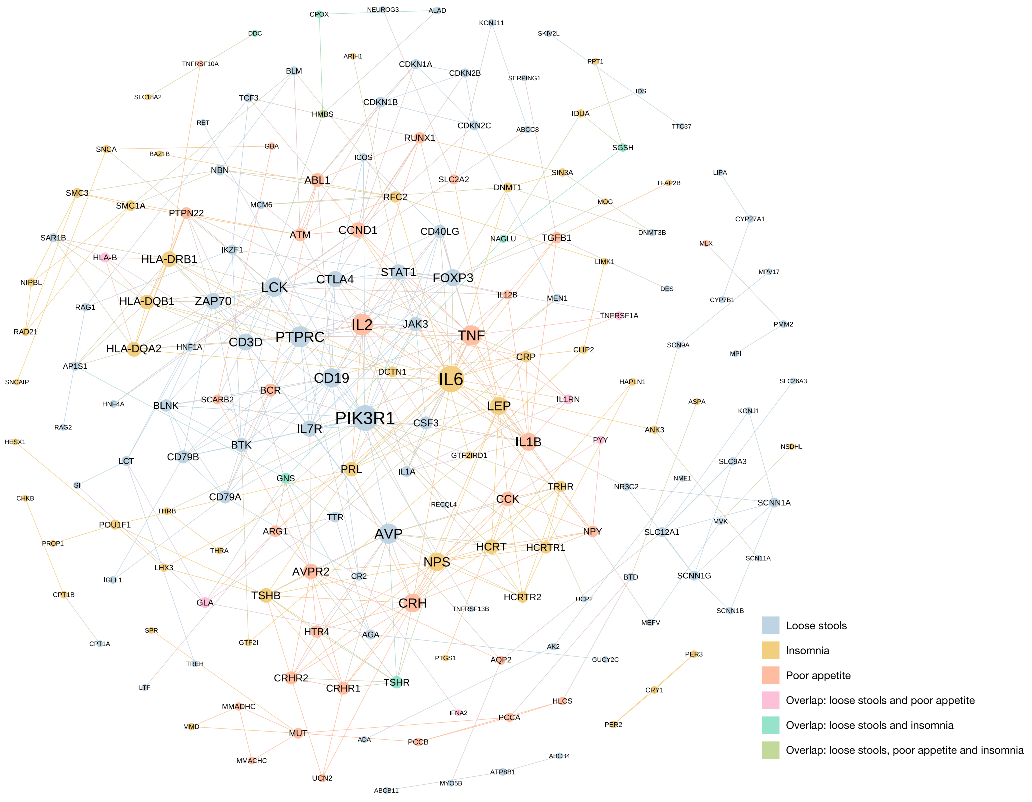

We extracted a PPI subnetwork of insomnia-loose stools-poor appetite symptom clusters which consisted of 183 nodes and 473 edges. The nodes represent the related genes of these symptoms in PPI network and edges represent the interactions of these genes in PPI network. Node size reflects the degree of symptom in the network (a high degree is represented by large node). Node color represents genes associated with different symptoms. PIK3R1, PTPRC and CD19 with higher degree in their associated PPI network, CD19 is a target of chimeric antigen receptor T-cells used in the treatment of lymphoblastic leukemia, which with a combination of constitutional symptoms and signs of bone marrow failure, such as anemia, thrombocytopenia and leukopenia[8]. We also found some targets (e.g. CNR1, CNR2 and CHRM1) in the network are related to gastrointestinal regulating drugs, including used for chemotherapy and postoperative drugs (e.g. dronabinol[10] and ramosetron[11]) and antidiarrheal agent (e.g. crofelemer[12] and furazolidone[13]).

Supplementary Figure 6. Construction of insomnia-night sweats-headache cluster of PPI network

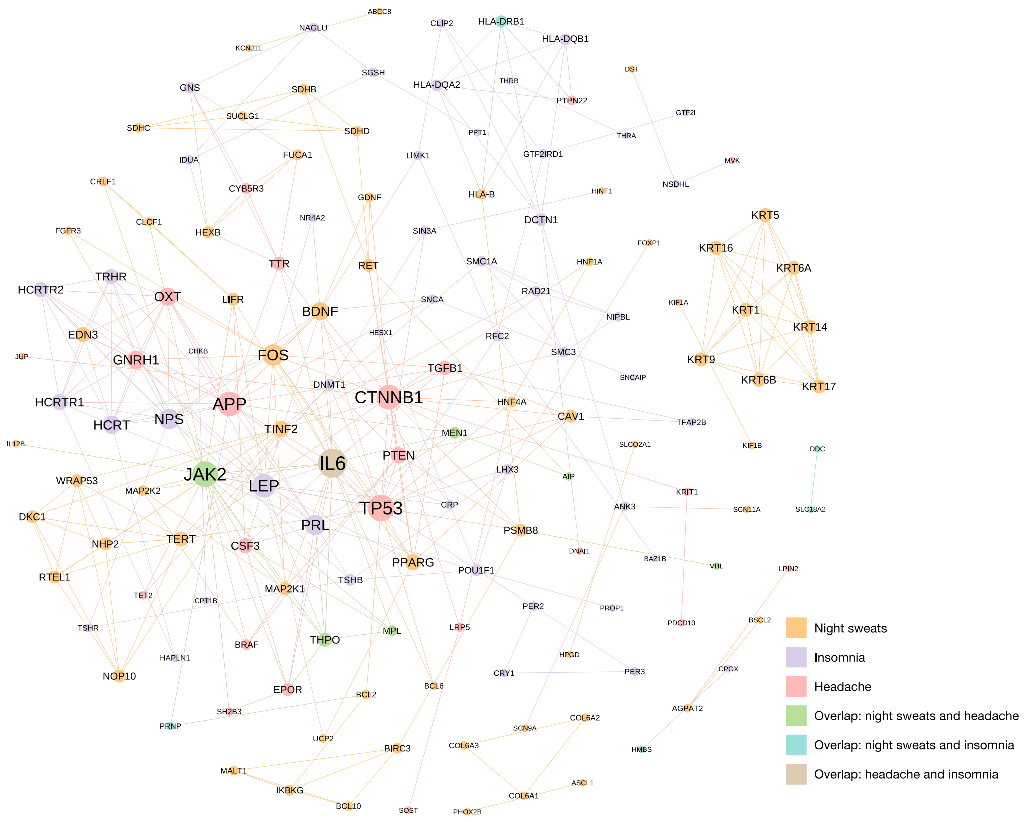

We extracted a PPI subnetwork of insomnia-night sweats-headache symptom clusters which consisted of 148 nodes and 344 edges. The nodes represent the related genes of these symptoms in PPI network and edges represent the interactions of these genes in PPI network. Node size reflects the degree of symptom in the network (a high degree is represented by large node). Node color represents genes associated with different symptoms. Insomnia-night sweats-headache cluster includes some genes with higher degree related headache in the network, such as CTNNB1, TP53 and APP, are involved in the development of tumors[14].The function of the overlapped genes related to the regulatory process of neurotransmitters (e.g. aminergic neurotransmitter loading into synaptic vesicle and positive regulation of nitric oxide biosynthetic process), whose imbalanced concentrations in the brain can cause symptoms such as headaches and insomnia[15,16].

Supplementary References

1 Chen, K., Xie, S. & Jin, W. Crucial lncRNAs associated with adipocyte differentiation from human adipose-derived stem cells based on co-expression and ceRNA network analyses. *PeerJ* **7**, e7544 (2019).

2 Luo, C.-l. *et al.* The effect of quercetin nanoparticle on cervical cancer progression by inducing apoptosis, autophagy and anti-proliferation via JAK2 suppression. *Biomedicine & pharmacotherapy* **82**, 595-605 (2016).

3 Carneiro, B. A. & El-Deiry, W. S. Targeting apoptosis in cancer therapy. *Nature reviews Clinical oncology* **17**, 395-417 (2020).

4 Spicer, A. P., Joo, A. & Bowling, R. A. A hyaluronan binding link protein gene family whose members are physically linked adjacent to chrondroitin sulfate proteoglycan core protein genes: the missing links. *Journal of Biological Chemistry* **278**, 21083-21091 (2003).

5 Abdolmaleky, H. M. *et al.* Epigenetic dysregulation of HTR2A in the brain of patients with schizophrenia and bipolar disorder. *Schizophrenia research* **129**, 183-190 (2011).

6 Tang, H., McGowan, O. O. & Reynolds, G. P. Polymorphisms of serotonin neurotransmission and their effects on antipsychotic drug action. *Pharmacogenomics* **15**, 1599-1609 (2014).

7 Refojo, D. *et al.* Glutamatergic and dopaminergic neurons mediate anxiogenic and anxiolytic effects of CRHR1. *Science* **333**, 1903-1907 (2011).

8 Terwilliger, T. & Abdul-Hay, M. Acute lymphoblastic leukemia: a comprehensive review and 2017 update. *Blood cancer journal* **7**, e577-e577 (2017).

9 Neumann, S. N. *et al.* Anemia and insomnia: a cross-sectional study and meta-analysis. *Chinese medical journal* **134**, 675 (2021).

10 Wong, B. S. *et al.* Randomized pharmacodynamic and pharmacogenetic trial of dronabinol effects on colon transit in irritable bowel syndrome- diarrhea. *Neurogastroenterology & Motility* **24**, 358-e169 (2012).

11 Rabasseda, X. Ramosetron, a 5-HT3 receptor antagonist for the control of nausea and vomiting. *Drugs of today (Barcelona, Spain: 1998)* **38**, 75-89 (2002).

12 Cottreau, J., Tucker, A., Crutchley, R. & Garey, K. W. Crofelemer for the treatment of secretory diarrhea. *Expert review of gastroenterology & hepatology* **6**, 17-23 (2012).

13 Arvind, S. & Shashi, R. Comparative study of furazolidone and furazolidone with phytoconstituentes on broiler chicken. *World Journal of Pharmaceutical Research* **5**, 1233-1241 (2016).

14 Gao, C. *et al.* Exon 3 mutations of CTNNB1 drive tumorigenesis: a review. *Oncotarget* **9**, 5492 (2018).

15 D'Andrea, G., Gucciardi, A., Perini, F. & Leon, A. Pathogenesis of cluster headache: from episodic to chronic form, the role of neurotransmitters and neuromodulators. *Headache: The Journal of Head and Face Pain* **59**, 1665-1670 (2019).

16 Olesen, J. The role of nitric oxide (NO) in migraine, tension-type headache and cluster headache. *Pharmacology & therapeutics* **120**, 157-171 (2008).
